# Downregulation of Alox5 Inhibits Ferroptosis to Improve Doxorubicin‐Induced Cardiotoxicity via the P53/SLC7A11 Pathway

**DOI:** 10.1111/jcmm.70641

**Published:** 2025-06-08

**Authors:** Wenxi Fang, Zhefu Hu, Bo Shen, Xiaofeng Zeng, Si Chen, Shasha Wang, Saiyang Xie, Wei Deng

**Affiliations:** ^1^ Department of Cardiology Renmin Hospital of Wuhan University Wuhan P.R. China; ^2^ Hubei Key Laboratory of Metabolic and Chronic Diseases Wuhan P.R. China; ^3^ Cardiovascular Research Institute of Wuhan University Wuhan China

**Keywords:** Alox5, doxorubicin, ferroptosis, P53, ROS

## Abstract

Doxorubicin (DOX) is an anthracycline chemotherapeutic drug used for tumour treatment. Due to DOX‐induced cardiotoxicity (DIC), its clinical application has been widely limited. Multiple studies have shown that ferroptosis is involved in the pathogenesis of DIC and that arachidonate 5‐lipoxygenase (Alox5) plays an important role in the occurrence and development of ferroptosis. The aim of this study was to provide evidence that silencing Alox5 alleviated DIC by affecting ferroptosis and identify mechanisms. Acute models of DIC were established in wild‐type (WT) C57BL/6 and Alox5‐deficient (Alox5 KO) mice and neonatal rat ventricular myocytes (NRVMs). Alox5 was upregulated in vivo and in vitro during DIC. Subsequently, we overexpressed the Alox5 gene in adult mice using a recombinant adenovirus expression vector (rAAV9). Compared with that in WT mice, overexpressing Alox5 accelerated DOX‐induced myocardial injury and cardiac dysfunction. This finding was also confirmed in vitro. In contrast, silencing the Alox5 gene protected against myocardial injury in the DIC model and reduced ferroptosis and inflammation, and this effect was confirmed in vitro. In addition, transcriptomics and GO enrichment analysis of adult mouse cardiomyocytes showed that Alox5 could ameliorate DIC by inhibiting ferroptosis and inflammation. Moreover, P53 was identified as a target of Alox5. Subsequently, in vivo and in vitro experiments showed that silencing Alox5 could alleviate ferroptosis and inflammation. Further in vivo and in vitro experiments demonstrated that dexrazoxane (DXZ) could ameliorate DIC caused by Alox5 overexpression by alleviating ferroptosis. Mechanistically, silencing Alox5 could reduce reactive oxygen species (ROS) production through the P53/SLC7A11 pathway. Furthermore, P53 inhibitors significantly inhibited the adverse effects of Alox5 overexpression on DIC. The final experiment showed that pharmacological inhibition of Alox5 could prevent DIC in vivo and in vitro. Our study showed that the downregulation of Alox5 alleviated myocardial damage associated with DIC via the P53/SLC7A11 pathway. Therefore, inhibiting Alox5 might be a potential strategy for the treatment of DIC.

**Abbreviations**
4‐HNE: 4‐Hydroxynonenal
Alox5: arachidonate 5‐lipoxygenase
CK‐MB: creatine kinase‐MB
CO: cardiac output
CSA: cross‐sectional area
cTNT: troponin T
CVD: cardiovascular disease
DIC: doxorubicin‐induced cardiotoxicity
DOX: doxorubicin
+dp/dt: maximal left ventricular pressure rising rate
−dp/dt: minimal rate of pressure decay
DXZ: dexrazoxane
Fer‐1: ferrostatin‐1
FPN: ferroportin
GPX4: glutathione peroxidase 4
GSH: glutathione
HW/TL: heart weight to tibia length
IL‐1β: interleukin‐1β
IL‐6: interleukin‐6
LDH: lactic acid dehydrogenase
LTB4: leukotriene B4
LVEDD: left ventricular end‐diastolic dimension
LVEF: left ventricle ejection fraction
LVESd: left ventricular end systolic diameter
LVFS: left ventricular fractional shortening
LVIDs: left ventricular end systolic diameter
MCP‐1: monocyte chemoattractant protein‐1
MDA: malonaldehyde
NADPH: nicotinamide adenine dinucleotide phosphate
NRVMs: neonatal rat ventricular myocytes
PTGS2: prostaglandin‐endoperoxide synthase 2
ROS: reactive oxygen species
SLC7A11: solute carrier family 7 member 11
STEAP3: Six‐transmembrane epithelial antigen of prostate 3
TFR: transferrin
TNF‐α: tumour necrosis factor‐α

## 1 Introduction

Doxorubicin‐induced cardiotoxicity (DIC) is the main concern for patients undergoing chemotherapy, and doxorubicin (DOX) chemotherapy results in left ventricular ejection fraction (LVEF) damage in 9% of patients treated each year [1]. DOX and related anthracycline drugs have significant therapeutic effects on leukaemia, breast cancer, lung cancer, lymphoma and other tumour diseases [2, 3, 4, 5]. Due to its cardiotoxic effects, including arrhythmia, ventricular dysfunction and heart failure, which could lead to poor prognosis and increased mortality, the clinical use of DOX has been widely limited [6]. At present, several antioxidants, such as vitamin E and N‐acetylcysteine, have been used to alleviate DIC. There are still many patients who are not satisfied with their therapeutic effects [7, 8]. Because various treatment strategies have reached a bottleneck, it is particularly urgent to explore new and more effective treatments for DIC to further improve the biological properties of the heart, inhibit cardiomyocyte death and ultimately curb the occurrence and development of cardiotoxic effects.

The pathogenesis of DIC includes cell death, inhibition of DNA/RNA/protein synthesis, increased reactive oxygen species (ROS) production, impaired energy metabolism, mitochondrial dysfunction, interstitial fibrosis, autophagy disorders and intracellular calcium homeostasis disorders [9]. Although there are many unknown factors associated with the pathogenesis of DIC, ferroptosis is a form of cell death, which includes the Fenton reaction, lipid peroxidation and ROS accumulation [10]. Lipid metabolism and ferroptosis: The oxidation of arachidonic acid (AA) is mainly regulated by acyl CoA synthase long chain family protein 4 (ACSL4), recombinant lysophosphatidylcholine acyltransferase 3 (LPCAT3) and the lipoxygenase protein family (LOXs). Furthermore, arachidonate 5‐lipoxygenase (Alox5) is an important branch of the lipoxygenase family [11]. In recent years, many studies have shown that ferroptosis is associated with various cardiovascular diseases (CVDs). Therefore, we need to understand its pathogenesis, which would help us identify potential therapeutic targets for CVDs and improve the treatment of CVDs [12].

Lipoxygenases are non‐heme iron‐containing oxidases that mainly include Alox5, Alox12 and Alox15 [13]. Previous studies have shown that Alox12 is required for p53‐mediated tumour suppression through a distinct ferroptosis pathway [14]. In addition, the absence of Alox15 in the mice heart could effectively alleviate ischaemia–reperfusion‐induced myocardial injury by inhibiting ferroptosis [15]. Alox5 is a key enzyme in the biosynthesis of chemical attractants and vasoactive leukotrienes, catalysing the production of ROS [16]. Alox5 is mainly present in white blood cells, mast cells, dendritic cells and B lymphocytes and is involved in cell membrane modification, lipid metabolism and redox balance [17]. The metabolite of polyunsaturated fatty acids that is oxidised by lipoxygenase is hydroxyeicosatetraenoic acid. Lipoxygenase plays an important role in inflammatory reactions, allergic reactions, lipid peroxidation and tumour growth [18]. When white blood cells are activated, AA is released from the nuclear membrane through the action of cytoplasmic phospholipase A2 and binds to the Alox5 activating protein (FLAP). The increase in Ca^2+^ concentrations in activated cells promotes the translocation of Alox5 to the nuclear membrane, where Alox5 obtains substrates from FLAP [19]. Our previous study showed that cardiomyocytes accumulated Alox5 in the nucleus in response to Ang‐II stimulation [20]. Alox5 is known to participate in various physiological and pathological processes. Alox5 has been studied in hepatitis, pancreatic cancer, asthma, Alzheimer's disease and other diseases [21, 22, 23, 24]. More importantly, there have been relevant studies on pulmonary hypertension, atherosclerosis, ischaemic heart disease, septic heart disease and pressure overload‐induced heart failure [20, 25, 26, 27, 28, 29]. In addition, Alox5 has been identified as an important regulator of ferroptosis that participates in the production of ROS [30]. However, the pathogenic role of Alox5 in DIC is still unclear. Therefore, our aim was to understand whether Alox5 is involved in DIC and the extent to which it causes myocardial damage and cardiac dysfunction after DOX stimulation.

In the current study, we explored the role of Alox5 in DIC and the expression and effects of Alox5 on mice and neonatal rat ventricular myocytes (NRVMs) in response to DOX stimulation. The role of Alox5 in DIC was investigated by genetic depletion of Alox5 and rAAV9‐mediated cardiac‐specific Alox5 overexpression. Through RNA sequencing and bioinformatics analyses, it was predicted that Alox5 could ameliorate DIC by inhibiting ferroptosis through the P53/SLC7A11 pathway. Subsequently, in vivo and in vitro experiments showed that silencing Alox5 could alleviate DIC. Furthermore, the ferroptosis inhibitor dexrazoxane (DXZ) ameliorated DIC caused by Alox5 overexpression by inhibiting ferroptosis. Additionally, silencing P53 could ameliorate DIC caused by Alox5 overexpression. In summary, Alox5 inhibited ferroptosis to ameliorate DIC through the P53/SLC7A11 pathway. Finally, pharmacological inhibition of Alox5 could prevent DIC in vitro and in vivo. Importantly, our study contributed to the understanding of the role of Alox5 in cardiomyocytes and demonstrated its potential as a therapeutic target for treating DIC.

## 2 Methods

### 2.1 Materials

Doxorubicin hydrochloride for injection (H44024359) was obtained from Shenzhen Wane Pharmaceutical Company. The IL‐1β mouse ELISA kit (#BMS6002), IL‐6 mouse ELISA kit (#KMC0061), TNF‐α mouse ELISA kit (#BMS607‐3), Pierce BCA protein assay kit (#23225), goat anti‐mouse IgG Alexa Fluor 568 secondary antibodies (#A11001), goat anti‐rabbit IgG Alexa Fluor 488 secondary antibodies (#A11011) and SlowFade gold antifade reagent with DAPI (#S36939) were purchased from Invitrogen (Carlsbad, CA, USA). Cardiac isoform of troponin T (cTnT) (H149‐4), Lactate dehydrogenase (LDH) (A020‐2‐2) and CK‐MB (H197‐1‐1) ELISA kits were purchased from Nanjing Jiancheng Bioengineering Institute (Nanjing, China). Ferrostatin‐1 (Fer‐1) (HY‐100579), DXZ (HY‐B0581) and zileuton (HY‐14164) were obtained from MedChem Express (Shanghai, China). 2,7‐Dichlorodihydrofluorescein diacetate (DCFH‐DA) (S0033S), dihydroethidium (DHE) (S0063), lipid peroxidation malondialdehyde (MDA) assay kit (S0131S), LDH release kit (C0016), cell counting kit‐8 (CCK‐8, #C0037), Lipo6000 transfection reagent (#C0526), GSH and GSSG detection kits (S0053), NADP+/NADPH detection kit (S0179) and nuclear and cytoplasmic protein extraction kit (P0028) were obtained from Beyotime Biotechnology (Shanghai, China). An Iron Colorimetric Assay Kit was obtained from APPLYGEN Company (Beijing, China). Anti‐rabbit/mouse EnVisionTM^+^/HRP antibodies for immunohistochemical staining were acquired from Gene Technology (Shanghai, China). A mouse leukotriene B4 (LTB4) ELISA kit (Ml201802‐C) was purchased from Shanghai mlbio. Pifithrin‐α (S2929) was purchased from Selleck.

### 2.2 Animals and Treatments

**FIGURE 1:**
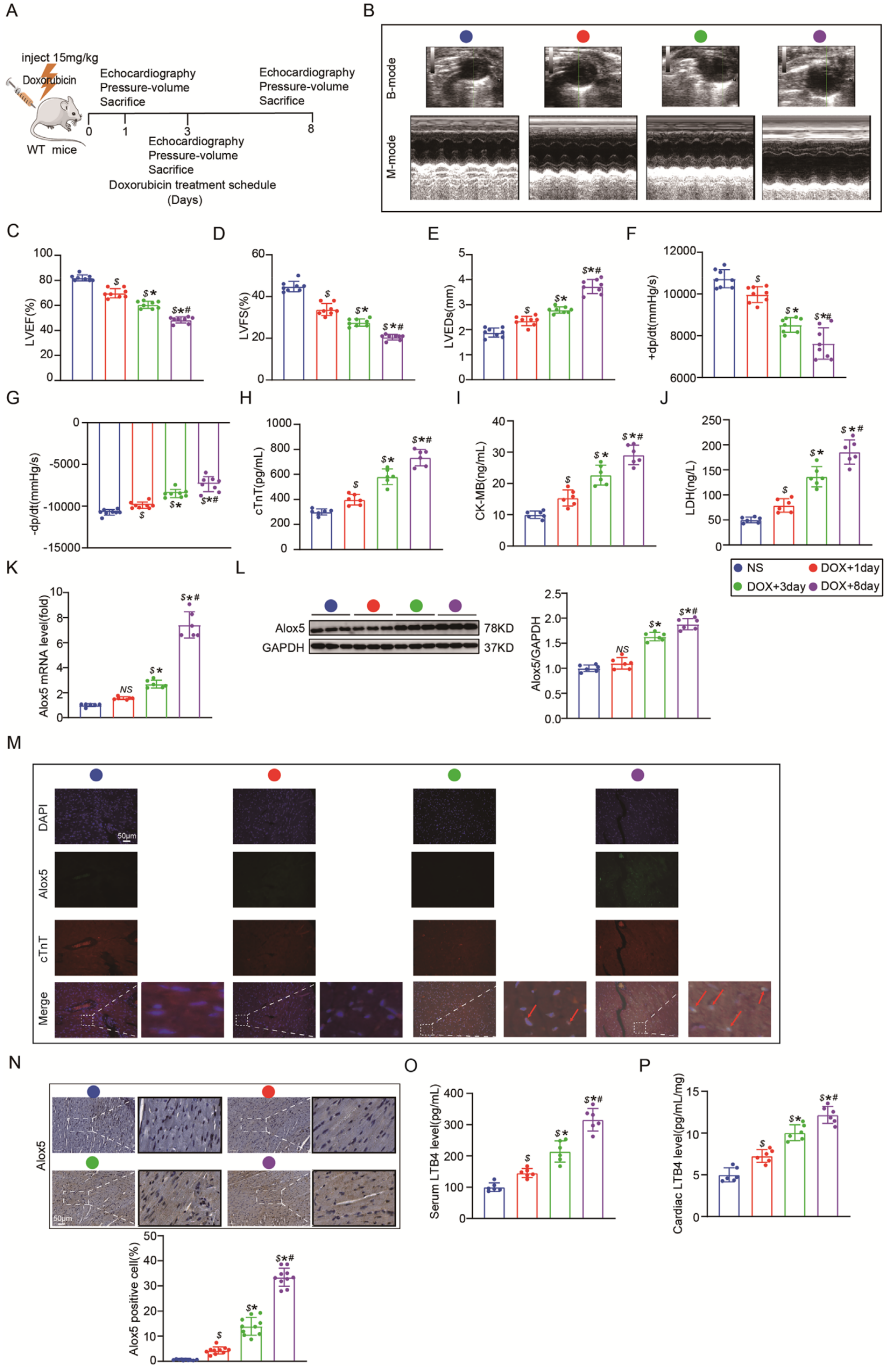
Alox5 expression is up‐regulated in DIC. (A) Schematic diagram of the acute mice modelling process of DIC. (B) Representative B‐ and M‐mode echocardiographic imaging of the heart in C57BL/6J mice group received NS or DOX (*n* = 8 per group). (C–E) Analysis of LVEF, LVFS and LVEDs in C57BL/6J mice group received NS or DOX (*n* = 8 per group), analysed by one‐way ANOVA with the Bonferroni post hoc test. (F,G) Analysis of +dp/dt and −dp/dt in C57BL/6J mice group received NS or DOX (*n* = 8 per group), analysed by one‐way ANOVA with the Bonferroni post hoc test. (H–J) Serum cTnT, CK‐MB and LDH levels in the C57BL/6J mice group received NS or DOX (*n* = 6 per group), analysed by one‐way ANOVA with the Bonferroni post hoc test. (K) qPCR analysis of Alox5 mRNA expression in C57BL/6J mice group received NS or DOX (*n* = 6 per group), analysed by one‐way ANOVA with the Bonferroni post hoc test. (L) Representative western blots and quantitation of Alox5 expression in heart tissue from C57BL/6J mice group received NS or DOX (*n* = 6 per group), analysed by one‐way ANOVA with the Bonferroni post hoc test. (M) Representative images of immunofluorescence staining of DAPI (blue), Alox5 (green), cTnT (red) and merge in C57BL/6J mice group received NS or DOX (*n* = 10 per group). (N) Representative images of immunohistochemistry staining of Alox5 and the quantitative results in C57BL/6J mice group received NS or DOX (*n* = 10 per group), analysed by one‐way ANOVA with the Bonferroni post hoc test. (O, P) Serum and cardiac LTB4 level in C57BL/6J mice group received NS or DOX (*n* = 6 per group), analysed by one‐way ANOVA with the Bonferroni post hoc test. Values represent mean ± SEM. NS *p* > 0.05: *p*‐value compared between DOX + 1 day group and NS group, $*p* < 0.05 indicated significantly different from NS group, *p < 0.05 indicated significantly different from DOX + 1 day group, #*p* < 0.05 indicated significantly different from DOX + 3 day group.

Alox5‐knockout (Alox5^−/−^) mice were purchased from Jackson Laboratory. Wild‐type (WT) male C57BL/6J mice (8–10 weeks) were purchased from the Chinese Academy of Medical Sciences (Beijing). Mice were anaesthetised with 3% pentobarbital sodium (50 mg/kg, Sigma) by intraperitoneal injection. Male mice were injected with DOX as shown in Figure 1A [31]. All animal experiments adhered to the guidelines for the Care and Use of Laboratory Animals (NIH publication number: 85‐23, revised 2011). Our experimental protocol was approved by the Guidelines for Animal Care and Use Committee of Renmin Hospital of Wuhan University (IvD number: WDRM.20210902B), which complied with the Guidelines for the Care and Use of Laboratory Animals published by the US National Institutes of Health. The animal experiments were conducted at the Cardiovascular Research Institute of Wuhan University. All experiments and data analysis were blindly performed by researchers who were unaware of the treatment allocations. As previously described [31], the mice were administered a single intraperitoneal injection of DOX (15 mg/kg) to generate the DIC model in vivo (Figure 1A). To induce exogenous expression of the Alox5 gene (encoding the Alox5 protein) in vivo, rAAV9‐Alox5 was used (Vi Gene Biosciences, Shandong, China), and the C57BL/6J mice were randomly divided into the control and experimental groups. As previously described [25], the experimental group was injected with ≥ 5 × 10^11 vg of recombinant rAAV9‐Alox5 in 200 μL of saline via the tail vein, while mice in the control group were injected with an equivalent volume of saline. Two weeks after viral delivery, western blot analysis was used to assess the overexpression efficiency in the heart. The mice were administered a single intraperitoneal injection of DOX (15 mg/kg) to induce DIC or an equal volume of normal saline (NS) as a control according to our previous study. Next, we proved that DXZ treatment ameliorated the deterioration of DIC caused by Alox5 overexpression by inhibiting ferroptosis. As previously described [32], the experimental group was injected with ≥ 5 × 10^11^ vg recombinant rAAV9‐Alox5 in 200 μL of saline via the tail vein, while mice in the control group were injected with an equivalent volume of saline. Two weeks after viral delivery, DXZ (20 mg/kg) was injected for 2 consecutive days [32]. Finally, the mice were administered a single intraperitoneal injection of DOX (15 mg/kg) to induce DIC or an equal volume of NS as a control according to our previous study [31]. Finally, as previously described, zileuton (100 mg/kg/day) was administered 2 weeks before DOX administration [25], and then the mice were administered a single intraperitoneal injection of DOX (15 mg/kg) to induce DIC or an equal volume of NS as a control [31]. Mice were euthanised using the cervical dislocation method.

### 2.3 Cells and Treatments

As previously described [20], NRVMs were isolated from the left ventricles of 1–3‐day‐old Sprague–Dawley rats using enzymatic digestion, and bromodeoxyuridine (100 μmol/L) was used to remove residual cardiac fibroblasts. Briefly, immunofluorescence (IF) analysis of α‐actinin was performed. Ten high magnification fields were randomly selected, and the number of positive cells and the total number of cells were counted. The purity of NRVMs was calculated as follows: positive cells/total number of cells × 100%. As previously described [33], NRVMs were cultured in the presence or absence of DOX (1 μmol/L) for 24 h. To show that Alox5 overexpression accelerated myocardial injury and cardiac dysfunction in DIC in vitro, as previously described [20], NRVMs were infected with an adenovirus encoding Alox5 (DesignGene Biotechnology, Shanghai, China) at an MOI of 50 for 12 h, and Ad‐CON was used as a control. Then, NRVMs were cultured in the presence or absence of DOX (1 μmol/L) for 24 h. Next, to show that Alox5 deletion attenuated myocardial injury and cardiac dysfunction in DIC in vitro, as previously described [25], small siRNA oligos (RiboBio, Guangzhou, China) were transfected with Lipo‐6000 reagent according to the manufacturer's instructions. Total RNA was extracted after 24 h to determine the effectiveness of siRNA transfection by immunoblotting. The Alox5 silencing targeted sequence was 5′‐GCATGACTTTGCTGACTTT‐3′, and the control targeted sequence was 5′‐GCTGCACAGAGTTGCCTAA‐3′ (RiboBio, Shanghai, China). After 8 h, NRVMs were cultured in the presence or absence of DOX (1 μmol/L) for 24 h. As previously described [34], NRVMs were infected with an adenovirus encoding Alox5 at an MOI of 50 for 12 h, and Ad‐CON was used as a control. Then, NRVMs were cultured in the presence or absence of DXZ (20 μmol/L) for 24 h. Finally, NRVMs were cultured in the presence or absence of DOX (1 μmol/L) for 24 h. As previously described [35], NRVMs were cultured in the presence or absence of zileuton (100 μmol/L) for 24 h. Then, NRVMs were cultured in the presence or absence of DOX (1 μmol/L) for 24 h. Finally, to prove that Alox5 could ameliorate DIC by inhibiting ferroptosis through the P53/SLC7A11 pathway, as previously described [36], NRVMs were infected with adenovirus encoding Alox5 at an MOI of 50 for 12 h, and Ad‐CON was used as a control. Then, after being pretreated with PBS or pifithrin‐α (20 μmol/L) for 12 h, NRVMs were cultured in the presence or absence of DOX (1 μmol/L) for 24 h.

### 2.4 Echocardiography and Haemodynamic Measurements

As previously described [37], echocardiography was performed following DOX treatment for 8 days. Mice were anaesthetised with 3% pentobarbital sodium (50 mg/kg, Sigma) by intraperitoneal injection and underwent echocardiography using the MyLab 30CV system. Left ventricular (LV) morphology was detected for 5 consecutive cardiac cycles, including measurements of left ventricular end systolic diameter (LVEDs), left ventricular end diastolic diameter (LVEDd), left ventricular fraction shortening (LVFS), and LVEF. For haemodynamic measurements, the anaesthetised mice were fixed, and bilateral subcostal incisions were performed. The diaphragm was exposed to the heart. A catheter (SPR‐839, Millar Instruments, Houston, TX, USA) was inserted into the left ventricle through the apical approach. As mentioned previously, haemodynamics were measured using cardiac catheterisation. The Millar Pressure–Volume System (MPVS‐400, Millar Instruments) was used to detect haemodynamic parameters such as the maximum pressure occurrence rate (+dP/dt max), minimal rate of pressure decay (‐dP/dt min) and cardiac output (CO). Mice were euthanised using the cervical dislocation method. Notably, LVFS was calculated with the following formula: LVFS = (LVIDd − LVIDs) × 100/LVIDd. LVEF was also evaluated using the Teichholtz formula: LVEF = ([100 − Y] × 0.15) + Y, Y = (LVIDd^2^ − LVIDs^2^) × 100/LVIDd^2^.

### 2.5 Tissues IF Staining

As previously described [20], place the prepared frozen sections of mouse myocardial tissue in an oven at 37°C for half an hour. After removal, place the myocardial tissue in a container containing PBS solution for 5 min each time, a total of three times. Place the myocardial tissue in a black and opaque wet box. Firstly, prepare a 100% hydrogen peroxide solution into a 3% hydrogen peroxide solution, then use a pipette to drip hydrogen peroxide onto the myocardial tissue. After 20 min, wipe off the 3% hydrogen peroxide solution on the surface of the myocardial tissue. Then, place the myocardial tissue slices into a container containing PBS solution for 5 min each time, a total of three times, and place them on a shaker. Firstly, thaw the sheep serum stock solution placed in a −20°C refrigerator, then prepare the thawed 100% sheep serum into 10% sheep serum. Then, use a pipette to drip 10% sheep serum onto the myocardial tissue. After 15 min, wipe off the 10% sheep serum on the surface of the myocardial tissue. Then, place the myocardial tissue slices into a container containing PBS solution for 5 min each time, a total of three times, and place them on a shaker. Wipe off the liquid on the myocardial tissue slice, dilute the corresponding primary antibody with PBS solution in a 1:100 ratio, and use a pipette to add 50 μL diluted the first antibody solution with drops to each myocardial tissue, then seal the black box and place it overnight in a refrigerator at 4°C. Place the black box containing myocardial tissue slices from the previous day at 37°C for at least half an hour of rewarming, drying the primary antibody solution on the myocardial tissue, place the myocardial tissue slices in a container containing PBS solution for 5 min each time, a total of three times, and place them on a shaker. Dry the liquid on the myocardial tissue, dilute the corresponding secondary antibody with PBS solution in a ratio of 1:200, and use a pipette to add 50 μL secondary antibody solution drops to each myocardial tissue, then seal the black box and incubate the secondary antibody for 1 h. The above operations need to be done in a dark place. Dry the secondary antibody solution on the myocardial tissue, place the myocardial tissue slices into a container containing PBS solution for 5 min each time, a total of three times, and place them on a shaker. Dry the PBS solution on the myocardial tissue, add DAPI dropwise to each myocardial tissue, and seal the slide with a cover glass. Observation and photography under a fluorescence microscope. All images were analysed by a person who was blinded to the treatments by using ImageJ.

### 2.6 Haematoxylin and Eosin Staining

As previously described [20], placing the corresponding group of myocardial tissue paraffin sections in a 55°C oven for at least 30 min to bake. Removing the corresponding group of myocardial tissue paraffin sections from the oven and placing them in a fresh xylene staining cylinder in sequence. The myocardial tissue paraffin sections could be completely immersed in xylene solution for 5 min each time, a total of three times. After the dewaxing was completed, placing the corresponding myocardial tissue paraffin sections in fresh anhydrous ethanol solution, fresh 95% ethanol solution, fresh 70% ethanol solution, and ddH_2_O in sequence. They could be completely immersed in fresh anhydrous ethanol, fresh 95% ethanol solution, fresh 70% ethanol solution, and ddH_2_O, and fresh anhydrous ethanol and ddH_2_O for 5 min each time, twice in total. Fresh 95% ethanol solution and fresh 70% ethanol solution, each time for 5 min, once in total. Placing the above‐mentioned myocardial tissue slices in a staining cylinder containing haematoxylin for 5 min, then taking out the myocardial tissue slices and placing them in a staining cylinder containing tap water. Turning on the faucet and rinsing the staining cylinder containing myocardial tissue slices for 10 min. Placing the above‐mentioned myocardial tissue slices in a staining cylinder containing 1% hydrochloric acid alcohol for 1–3 s, then taking out the myocardial tissue slices and placing them in a staining cylinder containing tap water. Turning on the faucet and rinsing the staining cylinder containing myocardial tissue slices for 10 min. After placing the above‐mentioned myocardial tissue slices in a staining cylinder containing Scott's solution for 1 min, then taking out the myocardial tissue slices and placing them in a staining cylinder containing tap water. Turning on the faucet and rinsing the staining cylinder containing myocardial tissue slices for 10 min. Placing the washed myocardial tissue slices into fresh anhydrous ethanol solution in sequence fresh 95% ethanol solution, fresh 70% ethanol solution, and ddH_2_O. Myocardial tissue slices could be completely immersed in fresh 70% ethanol, fresh 95% ethanol solution, fresh anhydrous ethanol, and fresh xylene. Fresh anhydrous ethanol and fresh xylene were immersed for 3 min each time, a total of twice. Fresh 95% ethanol solution and fresh 70% ethanol solution were immersed for 3 min each time, a total of once. The above‐mentioned myocardial tissue slices were sealed with neutral resin, preferably before xylene was dried, and the myocardial tissue slices were placed in a fume hood for air drying. After the myocardial tissue slices were air dried, they were observed and photographed under a light microscope. All images were analysed by a person who was blinded to the treatments by using ImageJ.

### 2.7 Immunohistochemical Staining

As previously described [20], placing the prepared frozen sections of mouse myocardial tissue in an oven at 37°C for half an hour. After removal, placing the myocardial tissue in a container containing PBS solution for 5 min each time, a total of three times. Placing the myocardial tissue in a black and opaque wet box. First, preparing a 100% hydrogen peroxide solution into a 3% hydrogen peroxide solution, then using a pipette to drip hydrogen peroxide onto the myocardial tissue. After 20 min, wiping off the 3% hydrogen peroxide solution on the surface of the myocardial tissue. Then, placing the myocardial tissue slices into a container containing PBS solution for 5 min each time, a total of three times, and placing them on a shaker. Firstly, thawing the sheep serum stock solution placed in a −20°C refrigerator, then preparing the thawed 100% sheep serum into 10% sheep serum. Then, using a pipette to drip 10% sheep serum onto the myocardial tissue. After 15 min, wiping off the 10% sheep serum on the surface of the myocardial tissue. Then, placing the myocardial tissue slices into a container containing PBS solution for 5 min each time, a total of three times, and placing them on a shaker. Wiping off the liquid on the myocardial tissue slice, diluting the corresponding primary antibody with PBS solution in a 1:100 ratio, and using a pipette to add 50 μL the diluted primary antibody solution to each myocardial tissue. Then, sealing the black box and placing it overnight in a refrigerator at 4°C. Placing the black box containing myocardial tissue slices from the previous day at 37°C for at least half an hour of rewarming, drying the primary antibody solution on the myocardial tissue, placing the myocardial tissue slices in a container containing PBS solution for 5 min each time, a total of three times, and placing them on a shaker. Drying the liquid on the myocardial tissue and diluting the corresponding secondary antibody with PBS solution in a ratio of 1:200. Using a pipette to add 50 μL the diluted secondary antibody solution to each myocardial tissue, then sealing the black box and incubating the secondary antibody for 1 h. The above operations needed to be done in a dark place. Drying the secondary antibody solution on the myocardial tissue, placing the myocardial tissue slices into a container containing PBS solution for 5 min each time, a total of three times, and placing them on a shaker. Drying the PBS solution on the myocardial tissue, adding DAPI dropwise to each myocardial tissue, and sealing the slide with a cover glass. Observation and photography under a fluorescence microscope. All images were analysed by a person who was blinded to the treatments by using ImageJ.

### 2.8 Western Blotting Analysis

As previously described [20], putting the reagent box equipped with sodium dodecyl sulfate polyacrylamide gel at room temperature, then washing the glass plate and the 15 holes comb, aligning the glass plate first, adding ddH_2_O into the pipette gun to detect whether there was water leakage between the glass plates, and configuring separation gel and concentrated gel according to the instructions of the reagent box. Firstly, using a measuring cylinder to measure 1800 mL of ddH_2_O and 200 mL of 10x SDS, and pouring them into a beaker. Stirring thoroughly with a glass rod to form 1x SDS. When sampling, adding 3 μL Marker and 3 μL Loading Buffer to the first well, and adding 6 μL of fresh protein samples extracted above to the other 12 wells. The electrophoresis inner liquid was 1 × SDS, and the electrophoresis outer liquid was electrophoresis waste liquid. Turning on the power supply of the electrophoresis instrument and adjusting the voltage to 75 V. When the sodium dodecyl sulfate polyacrylamide gel ran to the separation gel, adjusting the voltage of the electrophoresis instrument to 105 V. After the electrophoresis was completed, cutting the PVDF film into a box containing methanol, then removing the bubbles in the transfer tank, and adding fresh transfer solution to the transfer tank. Transferring the membrane according to the transfer parameters of 200 mA/2 blocks for 100 min. After the membrane transferred, sealing with a sealing solution prepared with fresh milk for 2 h. After sealing, washing the PVDF membrane with fresh TBST solution for 5 min each time, a total of three times. Incubating the corresponding PVDF membrane with a 1:1000 primary antibody solution as required, and placing it overnight on a shaker in a refrigerator at 4°C. The next day, washing the corresponding PVDF membrane with fresh TBST solution for 5 min each time, a total of three times. Incubating the corresponding PVDF membrane with a 1:10000 secondary antibody solution as required, and incubating for 2 h. Washing the corresponding PVDF membrane with fresh TBST solution for 5 min each time, a total of three times. Then, thoroughly mixing solution A and solution B in a 1:1 ratio with ECL developer solution, placing the corresponding PVDF film in the ECL developer solution, and using the corresponding chemical scanner to scan the film for colour development. The total protein levels were normalised to GAPDH/β‐actin/β‐Tubulin, and nuclear protein levels were normalised to Lamin B1. All images were analysed by a person who was blinded to the treatments by using ImageJ. All primary antibodies are listed in Table S1.

### 2.9 Quantitative Real‐Time Polymerase Chain Reaction

As previously described [20], to examine the mRNA expression of ferroptosis and inflammation markers, total RNA was collected from heart tissue and NRVMs using TRIzol reagent (Invitrogen, 15596‐026) and reverse‐transcribed to cDNA. qPCR was performed using a LightCycler 480 and SYBR Green Master Mix (cat. Number 04896866001, Roche). All primer details are listed in Table S2. The mRNA data were normalised to GAPDH.

### 2.10 Transcriptomic Profiling and Analysis

As previously described [20], selecting 6 fresh mouse heart tissue samples, with 3 samples from each of the DOX and DOX + Ko‐Alox5 groups. Sample RNA quality control and library construction results showed that the total amount, concentration, purity and integrity of the sample RNA all met the requirements of database construction. The agarose gel electrophoresis results showed that the size of the main segments of the RNA library was between 300 and 500 bp. The sequencing data volume of each sample reached 6G, the Q30 of each sample after Clean was more than 99%, and the proportion of clean reads was more than 93%. Clean data comparison of reference genomes showed that the mapping ratio was above 97%, indicating a high alignment rate. Reads were mainly distributed in the CDS region on the reference genome, which conformed to the typical characteristics of transcriptome sequencing results. Only significant enrichments with a Benjamini Hochberg‐adjusted *p*‐value less than 0.01 were considered. For heatmaps, leading edge gene sets were extracted from selected enriched pathways, annotated with their respective TPM values, converted into raw Z scores, and imported into GraphPad Prism 9.0 (GraphPad Software, USA) for heatmap generation.

### 2.11 IF Cell Staining

As previously described [20], using a pipette to add the corresponding NRVMs mentioned above to the cell slides, and after the corresponding treatment, waiting for the NRVMs to adhere to the cell slides. Using a pipette to remove the solution from the cell slides, and then using a pipette to drip the prepared PBS solution onto the cell slides for 5 min each time, a total of three times, and placing it on a shaker. First, preparing a 100% hydrogen peroxide solution into a 3% hydrogen peroxide solution, and then using a pipette to drip hydrogen peroxide onto the cell slide for 5 min each time, a total of three times, and placing it on a shaker. Using a pipette to remove the 3% hydrogen peroxide solution from the cell slides, and using a pipette to drip the prepared PBS solution onto the cell slides for 5 min each time, a total of three times, and placing it on a shaker. Firstly, thawing the sheep serum stock solution placed in a −20°C refrigerator, then preparing the thawed 100% sheep serum as 10% sheep serum, and then using a pipette to add 10% sheep serum to the cell slides. After 15 min, using a pipette to remove the 10% sheep serum from the surface of the cell slides. Finally, using a pipette to add the prepared PBS solution to the cell slides, each time for 5 min, a total of three times, and placing it on a shaker. Using a pipette to remove the liquid from the cell slides, diluting the corresponding primary antibody with PBS solution in a 1:100 ratio, and using a pipette to add 50 μL of the diluted primary antibody solution to each cell slide. Then, sealing the black box and placing it overnight in a refrigerator at 4°C. Placing the black box containing cell slides from the previous day at 37°C for at least half an hour of rewarming, using a pipette to remove the primary antibody solution from the cell slides, and using a pipette to drip the prepared PBS solution onto the cell slides for 5 min each time, a total of three times, and placing it on a shaker. Using a pipette to aspirate the PBS solution from the cell slides, diluting the corresponding secondary antibody with PBS solution in a ratio of 1:200, and using a pipette to add 50 μL of the diluted secondary antibody solution to each cell slide. Then, sealing the black box and incubating the secondary antibody for 1 h. The above operations needed to be done in a dark place. Using a pipette to remove the secondary antibody solution from the cell slides. For each cell slide, using a pipette to drip PBS solution for 5 min each time, a total of 3 times, and placing it on a shaker. Using a pipette to remove PBS solution from the cell slides, adding DAPI dropwise to each slide, and covering the cell slides with DAPI. Observation and photography under a fluorescence microscope. All images were analysed by a person who was blinded to the treatments by using ImageJ.

### 2.12 DHE Staining

As previously described [37], placing the prepared frozen sections of mouse myocardial tissue in an oven at 37°C for half an hour. After removal, placing the myocardial tissue in a container containing PBS solution for 5 min each time, a total of three times. Placing the myocardial tissue in a black and opaque wet box. Wiping off the PBS solution from the myocardial tissue slice, using a pipette to add 100 μL of cleaning solution working solution onto the myocardial tissue slice, and incubating for 10 min. Wiping off the cleaning solution working solution from the myocardial tissue slice, using a pipette to drip 50 μL of staining working solution onto the myocardial tissue slice, closing the black box, and incubating in a 37°C oven for 1 h. Wiping off the staining solution from the myocardial tissue slice, placing the myocardial tissue slice in a container containing PBS solution for 5 min each time, a total of three times, and placing it on a shaker. Drying the PBS solution on the myocardial tissue, adding DAPI dropwise to each myocardial tissue, and sealing the slide with a cover glass. Observation and photography under a fluorescence microscope. Quantification was performed with ImageJ.

### 2.13 DCFH‐DA Staining

As previously described [37], diluting DCFH‐DA with serum‐free culture medium at a ratio of 1:1000 to a final concentration of 10 μmol/L. Removing the cell culture medium and adding diluted 1 mL DCFH‐DA to one well of the six‐well plate. Incubating in a 37°C cell culture incubator for 20 min. Washing the cells three times with serum‐free cell culture medium to fully remove DCFH‐DA that had not entered the cells. Finally, cells were visualised in a blinded manner with an Olympus IX53 fluorescence microscope. Quantification was performed with ImageJ.

### 2.14 Cell Nuclear Proteins Exaction

As previously described [20], removing the reagents from the reagent kit from the −20°C refrigerator for rewarming, and using a pipette to add PMSF into the EP tube containing cytoplasmic protein extraction reagent A. Then, precooling PBS solution was added to the corresponding processed NRVMs culture dish, and all NRVMs were scraped off as much as possible with a scraper. The precooling centrifuge was used to centrifuge the NRVMs solution, and the supernatant was discarded with a pipette, leaving behind cell precipitates. Using a pipette to add the freshly prepared cytoplasmic protein extraction reagent A to the EP tube containing cell precipitation and placing it on top of a vortex mixer for 1 min. Placing the EP tube into a box containing a large amount of ice. After 30 min, using a pipette to add cytoplasmic protein extraction reagent B to the aforementioned EP tube and placing it on top of a vortex mixer for 1 min. Finally, placing the EP tube into a box containing a large amount of ice for 30 min. Precooling centrifuge, with centrifugation parameters set to 12000r 4°C, and centrifugation for 10 min. After centrifugation, using a pipette to aspirate the supernatant into a new EP tube of the corresponding composition. Adding nuclear protein extraction reagent to the EP tube containing the remaining precipitate, and placing it on a vortex mixer for 1 min. Then putting it back into a box containing a large amount of ice for 5 min and repeating the cycle 8 times. Finally, precooling the centrifuge and centrifuging for 10 min under the parameter setting of 12000r 4°C. Then, pipetting the supernatant into the corresponding group of EP tubes, which is the NRVMs nucleus protein.

### 2.15 LTB4 Measurement

As previously described [25], using 1%–2% isoflurane for inhalation anaesthesia in mice, gently stroking the skin of the whole body to relax, and then using one hand to press on both sides of the mouse's neck, causing congestion of the orbital venous plexus. Then, with the other hand, holding a capillary glass tube and inserting it into the venous plexus in the fundus of the eye, causing the venous blood to slowly flow out. After collecting the required amount of blood, stopping the bleeding with a sterile dry cotton ball and removing the hand. Stopping bleeding and observing for 10 min. Only after the mouse had no bleeding could it be placed back in the cage. The mouse leukotriene B4 (LTB4) ELISA kit was used to determine the content of LTB4 in plasma. Diluting the collected peripheral blood serum sample with a sample diluent of 1:1, and then introducing 50 μL of the diluted sample into the reaction. Subsequently, 50 μL biotin labelled LTB4 antibody was added after 80 μL affinity streptomycin HRP. Then, after terminating the reaction, the OD value at 450 nm was detected, and the LTB4 level was calculated using the standard curve. The levels of LTB4 in serum and cardiac tissue were detected by colorimetric assays using kits. The samples were read at 450 nm.

### 2.16 Biochemical Analysis

As previously described, serum levels of LDH, cTnT, interleukin‐1β (IL‐1β), tumour necrosis factor‐α (TNF‐α), interleukin‐6 (IL‐6) and creatine kinase isoenzymes (CK‐MB) were detected by an automatic biochemical analyser (ADVIA 2400, Siemens) [38, 39]. The operation method was the same as the LTB4 measurement. The samples were read at 450 nm.

### 2.17 Cell Counting Kit‐8 (CCK‐8)

As previously described [32], we used a CCK‐8 assay kit to evaluate the viability of treated cells. A 100 μL NRVMs suspension was dispensed in a 96‐well plate, and the cells were subjected to the different treatments according to the experimental design. Then, 10 μL of CCK‐8 solution was added to the treated cells and incubated for 4 h. Cell viability was measured by determining the absorbance at a wavelength of 450 nm with a microplate reader and was calculated as follows: cell viability (%) = OD treatment/OD control.

### 2.18 Non‐Heme Iron in Serum and Cardiac Tissue

As previously described [32], precooling the high‐speed tissue grinder to 4°C, accurately weighing fresh mouse heart tissue, cutting it into small pieces with scissors, adding 10 mg/100 μL lysis solution to the heart sample, symmetrically placing the EP tube containing the myocardial tissue sample in the grinder, and grinding it at a frequency of 50 Hz was performed. After grinding for 200 s, using a pipette to transfer the freshly ground myocardial tissue homogenate into the corresponding new EP tube, and using tweezers to recover the ground steel balls into the EP tube containing anhydrous ethanol were done. The next step was to place the EP tube containing mouse myocardial tissue homogenate on top of a box containing a large amount of ice. The myocardial tissue homogenate was lysed 30 times on the ice box using an ultrasonic lysis instrument (5 kHz), and then precooled in a high‐speed centrifuge. The EP tube containing mouse myocardial tissue homogenate was symmetrically placed in the centrifuge, and the centrifugation parameters were set to 12000r for 40 min. After centrifugation, a pipette was used to aspirate the supernatant into the corresponding new EP tube. Using a pipette to aspirate buffer solution and 4.5% potassium permanganate solution were aspirated separately into an EP tube and mixed thoroughly to form mixture A. Then, the corresponding standard was prepared according to the instructions. The blank control group in the EP tube consisted of 100 μL dilution solution and 100 μL mixture A aspirated with a pipette. The diluted standard group in the EP tube consisted of 100 μL mixture A and 100 μL standard working solution. The sample group split in the EP tube consisted of 100 μL mixture A and 100 μL sample. The EP tube of the above sample was incubated in a 55°C oven for 1 h, then cooled to room temperature. Droplets on the tube cover and walling were centrifuged into the bottom of the tube. Then, 30 μL of iron ion detection agent was added to the corresponding EP tube using a pipette. After incubation at room temperature for 30 min, 200 μL of the supernatant was taken using a pipette and placed on a 96 well plate. The levels of non‐heme iron in serum and cardiac tissue were detected by colorimetric assays using kits. The Fe^2+^ concentration was measured and analysed at a wavelength of 550 nm.

### 2.19 MDA in Serum and Cardiac Tissue

As previously described [32], precooling the high‐speed tissue grinder to 4°C, accurately weighing fresh mouse heart tissue, cutting it into small pieces with scissors, adding 10 mg/100 μL lysis solution to the heart sample, symmetrically placing the EP tube containing the myocardial tissue sample in the grinder, and grinding it at a frequency of 50 Hz are essential steps. After grinding for 200 s, using a pipette to transfer the freshly ground myocardial tissue homogenate into the corresponding new EP tube, and using tweezers to recover the ground steel balls into the EP tube containing anhydrous ethanol are necessary actions. The next step was to place the EP tube containing mouse myocardial tissue homogenate on top of a box containing a large amount of ice. The myocardial tissue homogenate was lysed 30 times on the ice box using an ultrasonic lysis instrument (5 kHz), and then precooled in a high‐speed centrifuge. The EP tube containing mouse myocardial tissue homogenate was symmetrically placed in the centrifuge, and the centrifugation parameters were set to 12000r for 40 min. After centrifugation, using a pipette to aspirate the supernatant into the corresponding new EP tube is required. Adding the above‐mentioned cracking solution or PBS solution as a blank control by using a pipette to the newly formed EP tube, and then using a pipette to add the MDA detection working solution to the corresponding EP tube and shaking it thoroughly are critical tasks. Placing the EP tube in a boiling water bath for 15 min, and symmetrically placing the EP tube that had dropped to room temperature in a centrifuge with an intrinsic parameter of 1000r and centrifuged for 20 min are subsequent steps. After centrifugation, using a pipette to take the supernatant from the corresponding group of EP tubes and adding it to the corresponding 96 well plate is the final action. Finally, placing the 96 well plate on an enzyme‐linked immunosorbent assay (ELISA) reader with a wavelength of 532 nm to measure the concentration of MDA is performed.

### 2.20 LDH in the Cell Culture Medium

As previously described [32], filling the extracted NRVMs with a six‐well plate, and after NRVMs were completely attached to the wall, transfecting and stimulating them accordingly. Using a pipette to aspirate the above culture medium, then adding LDH release reagent to the pipette and culture in the cell culture box for 2 h. Adding the corresponding cell lysis solution and adding the six‐well plate to the corresponding ice to fully lyse it. After 30 min, using a pipette to blow thoroughly, and then placing it in a 1.5 mL EP tube. Precooling the centrifuge and symmetrically placing the corresponding group of EP tubes into the centrifuge. Centrifuging for 20 min under conditions of 12000r and 4°C. After centrifugation, using a pipette to absorb the supernatant as much as possible and adding it to the corresponding 96‐well plates. Using a pipette to add 60 μL of LDH detection working solution, incubating at room temperature for 1 h, and then measuring the absorbance at 490 nm.

### 2.21 Determination of GSH Levels

As previously described [25], according to the instructions of GSH and GSSH detection kits, preparing GSH clearance reagent working solution, total glutathione detection working solution, 5‐fold diluted glutathione reductase, NADPH reserve solution, protein removal reagent M solution, DTNB reserve solution, diluted GSH clearance assistance, 0.5 mg/mL NADPH, and GSSG reserve solution. Using a pipette to dilute 10 mM GSSG stock solution with protein removal reagent M solution to 15 μM GSSG solution. Precooling the high‐speed tissue grinder to 4°C, accurately weighing fresh mouse heart tissue, cutting it into small pieces with scissors, adding 10 mg/100 μL protein removal reagent M solution to the heart sample, symmetrically placing the EP tube containing myocardial tissue sample in the grinder, and grinding it at a frequency of 50 Hz. After grinding for 200 s, using a pipette to transfer the freshly ground myocardial tissue homogenate into the corresponding new EP tube, and using tweezers to recover the ground steel balls into the EP tube containing anhydrous ethanol. The next step was to place the EP tube containing mouse myocardial tissue homogenate on top of a box containing a large amount of ice. The myocardial tissue homogenate was lysed 30 times on the ice box using an ultrasonic lysis instrument (5 kHz), and then precooling in a high‐speed centrifuge. The EP tube containing mouse myocardial tissue homogenate was symmetrically placed in the centrifuge, and the centrifugation parameters were set to 12000r for 40 min. After centrifugation, using a pipette to aspirate the supernatant into the corresponding new EP tube. Adding samples or standards to a new 96 well plate, 150 μL total glutathione detection working solution, 50 μL 0.5 mg/mL NADPH, and incubating at room temperature for 10 min. Finally, adding 50 μL 0.5 mg/mL NADPH solution and gently shaking the 96 well plate left and right. Placing a 96 well plate on an enzyme‐linked immunosorbent assay (ELISA) reader to measure the total content of GSH, for a total of 5 times with a 10 min interval between each measurement. The samples were read at 412 nm.

### 2.22 Determination of NAPDH Levels

As previously described [32], precooling the high‐speed tissue grinder to 4°C, accurately weighing fresh mouse heart tissue, cutting it into small pieces with scissors, adding 10 mg/100 μL NADP^+^/NADPH extraction solution to the heart sample, symmetrically placing the EP tube containing the myocardial tissue sample in the grinder, and grinding it at a frequency of 50 Hz was performed. After grinding for 200 s, a pipette was used to transfer the freshly ground myocardial tissue homogenate into the corresponding new EP tube, and tweezers were used to recover the ground steel balls into the EP tube containing anhydrous ethanol. The next step was to place the EP tube containing mouse myocardial tissue homogenate on top of a box containing a large amount of ice. The myocardial tissue homogenate was lysed 30 times on the ice box using an ultrasonic lysis instrument (5 kHz), and then precooled in a high‐speed centrifuge. The EP tube containing mouse myocardial tissue homogenate was symmetrically placed in the centrifuge, and the centrifugation parameters were set to 12000r for 40 min. After centrifugation, a pipette was used to aspirate the supernatant into the corresponding new EP tube. NADPH standard and G6PDH working solution were prepared according to the instructions of the NADP^+^/NADPH detection kit. A pipette was used to suck 50 μL of the sample to be tested into a 96 well plate, and blank control wells, standard wells, and sample wells were set according to the instructions. The 96 well plate was incubated in a 37°C oven for 30 min, then a pipette was used to add 10 μL of colorimetric solution to each well, and incubated in the dark at 37°C for 10–20 min. At this time, an orange yellow formazan would form. The samples were read at 450 nm.

### 2.23 Statistical Analysis

All data in this study are presented as the mean ± standard error of the mean (SEM). In the animal and cell culture experiments, we used *n* ≥ 6 for biochemical analyses and *n* ≥ 10 for pathological experiments. All animal experiments were performed in a randomised and blinded manner. For all imaging analyses, an observer who was blinded to the experimental groups conducted the quantitation. No samples or animals were excluded from the analyses. Student's *t* test was applied for pairwise comparisons. For multiple group comparisons, one‐way or two‐way ANOVA with Tukey's post hoc test was performed. Differences were deemed statistically significant at *p* < 0.05. GraphPad Prism software (Version 9.0, GraphPad Software) was used for statistical analysis.

## 3 Results

### 3.1 Alox5 Expression Was Upregulated in DIC In Vivo and In Vitro

To examine DIC, we first created the mice models of acute DIC (Figure 1A), as previously described [31]. Echocardiography and haemodynamic analysis showed that compared with the NS group, the DOX group experienced more severe cardiac dysfunction with increasing DOX stimulation time, which was characterised by decreased LVEF, LVFS, +dp/dt and ‐dp/dt and increased LVEDs (Figure 1B–G). In addition, we examined the changes in myocardial zymogram after DOX administration and found that cardiac injury markers such as cTnT, CK‐MB, and LDH (Figure 1H–J) increased with the prolongation of stimulation time in the DOX group. To evaluate cardiac Alox5 expression following acute DIC, Alox5 mRNA and protein levels were measured in heart tissues. The qPCR results showed hyperactive Alox5 transcription in DIC hearts (Figure 1K). Consistently, Western blot analysis showed that the protein expression level of Alox5 began to increase on day 1 and peaked at 8 days (Figure 1L). Consistent with the immunoblot assays and qPCR results, the IF results showed that Alox5 expression in DIC hearts was significantly higher than that in control hearts, and Alox5 was located in the nucleus in mice cardiomyocytes (Figure 1M). In addition, immunohistochemical staining confirmed that the protein expression of Alox5 began to increase in DIC hearts on day 1 and peaked at 8 days (Figure 1N). As a lipid mediator produced by AA and a pro‐inflammatory mediator produced by the Alox5 enzyme, LTB4 is an essential chemoattractant of inflammatory leukocytes [40]. Subsequently, serum and cardiac LTB4 levels were detected, and DOX stimulation significantly upregulated serum and cardiac LTB4 levels, indicating a significant increase in Alox5 enzyme activity in DIC (Figure 1O,P). These results suggested that Alox5 expression was upregulated in DIC in vivo.

Similarly, we established the in vitro DIC model in NRVMs induced by DOX (Figure S1). According to our previous research, the optimal concentration of DOX in vitro was 1 μmol/L, as validated by CCK‐8 experiments (Figure S1) [31]. To evaluate Alox5 expression following acute DIC in vitro, Alox5 mRNA and protein levels were investigated in NRVMs. First, qPCR showed hyperactive Alox5 transcription in DOX‐induced NRVMs injury (Figure S1). Consistently, Western blotting confirmed that the protein expression of Alox5 increased with increasing DOX concentrations, and the highest expression level of Alox5 protein was observed in response to 1 μmol/L (Figure S1). In addition, we further analysed the subcellular localisation of Alox5 in NRVMs. Immunoblot analysis of isolated nuclear fractions or IF analysis was performed on NRVMs with or without DOX treatment, and we found that DOX treatment contributed to Alox5 accumulation in the nucleus (Figure S1). In summary, in vivo and in vitro experiments showed that Alox5 expression was upregulated in DIC.

### 3.2 Alox5 Overexpression Accelerated Myocardial Injury and Cardiac Dysfunction in DIC

**FIGURE 2:**
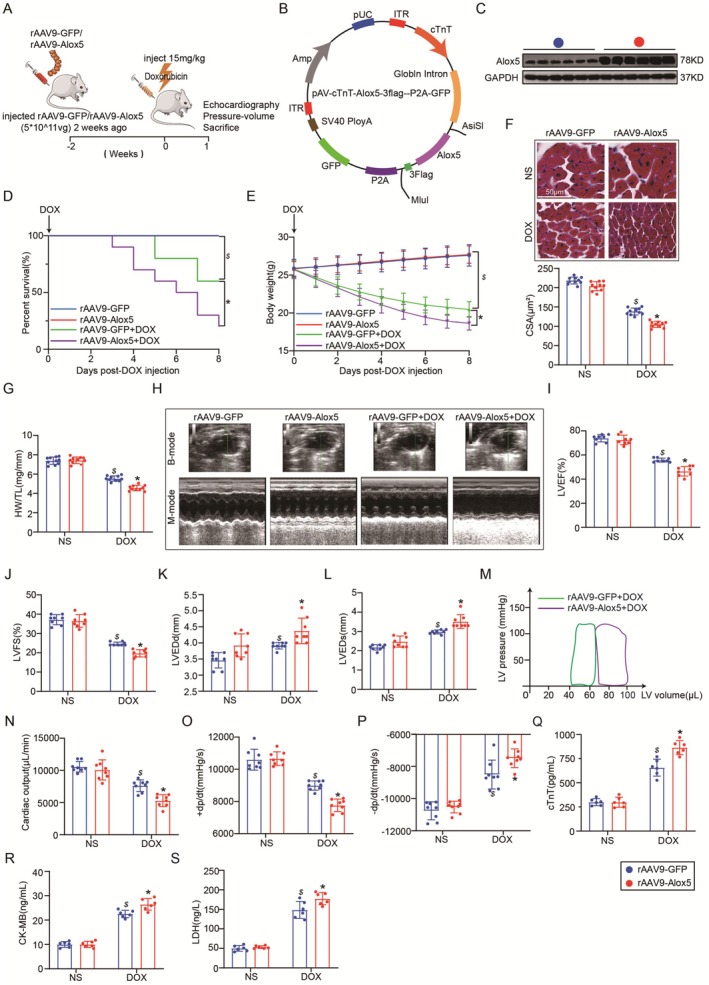
Alox5 overexpression accelerated myocardial injury and cardiac dysfunction in DIC. (A) Schematic diagram of the acute mice modelling process of DIC. (B) The plasmid for recombinant AAV9 vector overexpressing GFP or Alox5. (C) Representative western blots of Alox5 expression in rAAV9‐GFP or rAAV9‐Alox5 mice groups (*n* = 6 per group). (D) Survival analysis DOX 8 day in rAAV9‐GFP or rAAV9‐Alox5 mice group received NS or DOX (*n* = 10 per group). Comparison of survival curves between rAAV9‐GFP and rAAV9‐Alox5 mice groups using the log‐rank (Mantel‐Cox) test. (E) Body weight alterations in rAAV9‐GFP or rAAV9‐Alox5 mice groups received NS or DOX (*n* = 10 per group). (F) Representative HE staining images and the quantitative results in rAAV9‐GFP or rAAV9‐Alox5 mice group received NS or DOX (*n* = 10 per group), analysed by two‐way ANOVA with Tukey's post hoc test. (G) Statistical results of the heart weight/tibia length (HW/TL) in rAAV9‐GFP or rAAV9‐Alox5 mice group received NS or DOX (*n* = 10 per group), analysed by two‐way ANOVA with Tukey's post hoc test. (H) Representative B‐ and M‐mode echocardiographic imaging of the heart in rAAV9‐GFP or rAAV9‐Alox5 mice group received NS or DOX (*n* = 8 per group). (I–L) Analysis of LVEF, LVFS, LVEDd and LVEDs in rAAV9‐GFP or rAAV9‐Alox5 mice group received NS or DOX (*n* = 8 per group), analysed by two‐way ANOVA with Tukey's post hoc test. (M) Representative PV loops of rAAV9‐Alox5 + DOX or rAAV9‐GFP + DOX mice group (*n* = 8 per group), analysed by unpaired Student t‐test. (N–P) Analysis of cardiac output, +dp/dt and ‐dp/dt in rAAV9‐GFP or rAAV9‐Alox5 mice group received NS or DOX (*n* = 8 per group), analysed by two‐way ANOVA with Tukey's post hoc test. (Q–S) Serum cTnT, CK‐MB, and LDH levels in rAAV9‐GFP or rAAV9‐Alox5 mice groups received NS or DOX (*n* = 6 per group), analysed by Two‐way ANOVA with Tukey's post hoc test. The results are shown as mean ± SEM. $*p* < 0.05: *p*‐value compared between rAAV9‐GFP + DOX group and rAAV9‐GFP group, *p < 0.05: *p*‐value compared between rAAV9‐GFP + DOX group and rAAV9‐Alox5 + DOX group.

To assess the role of Alox5 in the heart in response to DOX stimulation, we first examined whether Alox5 supported or inhibited DIC in vivo. We overexpressed Alox5 (Figure 2A) by using rAAV9‐Alox5 (Figure 2B), which was administered to WT mice through a single tail vein injection. Two weeks later, Alox5 overexpression in the heart was confirmed by Western blotting (Figure 2C). First, the death rates of mice injected with rAAV9‐Alox5 were higher than those of control mice injected with rAAV9‐GFP after DOX treatment (Figure 2D). Furthermore, Alox5 overexpression accelerated body weight, the heart weight to tibia length (HW/TL) ratio and the cardiomyocyte CSA after DOX treatment (Figure 2E–G). Subsequently, the effects of Alox5 on cardiac function were further assessed by echocardiography and haemodynamic analysis. Importantly, we showed that Alox5 overexpression accelerated cardiac dysfunction in DIC and reduced LVEF, LVFS, CO, +dp/dt and ‐dp/dt and increased LVEDd and LVEDs (Figure 2H–P). Finally, we examined the alterations in the myocardial enzyme profile after DOX administration and found Alox5 overexpression accelerated cardiac injury such as cTnT, CK‐MB, and LDH (Figure 2Q–S). In summary, these experiments indicated that Alox5 overexpression accelerated DOX‐induced myocardial injury and cardiac dysfunction.

We assessed the role of Alox5 overexpression in DOX‐treated cardiomyocytes in vitro. We first overexpressed Alox5 through transfection with adenovirus (Ad)‐Alox5 or Ad‐CON (Figure S2A), as previously described [20]. Next, the efficiency of Alox5 gene transfer into NRVMs was evaluated by Western blotting after 12 h (Figure S2B). Furthermore, CCK‐8 experiments and LDH release assays showed a significant decrease in cell viability and an increase in extracellular LDH levels in Ad‐Alox5 NRVMs subjected to DOX, suggesting that Alox5 overexpression accelerated cardiomyocyte death in vitro in response to DOX stimulation (Figure S2C,D). The in vivo and in vitro experiments showed that Alox5 overexpression could accelerate DOX‐induced myocardial injury and cardiac dysfunction.

### 3.3 Alox5 Deletion Attenuated Myocardial Injury and Cardiac Dysfunction in DIC

**FIGURE 3:**
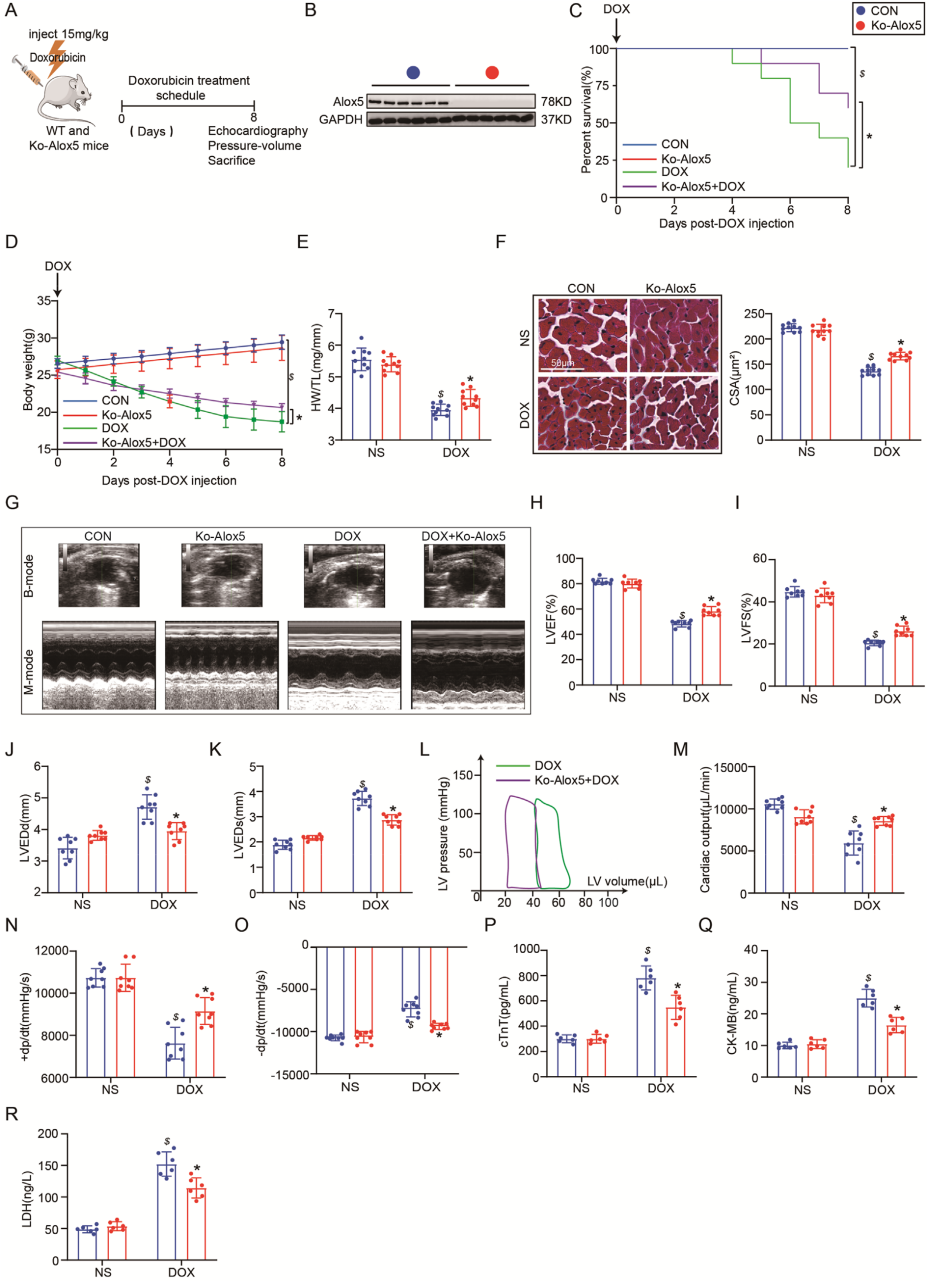
Alox5 deletion attenuated myocardial injury and cardiac dysfunction in DIC. (A) Schematic diagram of the acute mice modelling process of DIC. (B) Representative western blots of Alox5 expression in CON or Ko‐Alox5 mice groups (*n* = 6 per group). (C) Survival analysis DOX 8 day in CON or Ko‐Alox5 mice group received NS or DOX (*n* = 10 per group). Comparison of survival curves between DOX and Ko‐Alox5 + DOX mice group using the log‐rank (Mantel–Cox) test. (D) Body weight alterations in CON or Ko‐Alox5 mice groups received NS or DOX (*n* = 10 per group). (E) Statistical results of the heart weight/tibia length (HW/TL) in CON or Ko‐Alox5 mice group received NS or DOX (*n* = 10 per group), analysed by two‐way ANOVA with Tukey's post hoc test. (F) Representative HE staining images and the quantitative results in CON or Ko‐Alox5 mice groups received NS or DOX (*n* = 10 per group), analysed by Two‐way ANOVA with Tukey's post hoc test. (G) Representative B‐ and M‐mode echocardiographic imaging of the heart in CON or Ko‐Alox5 mice group received NS or DOX (*n* = 8 per group). (H–K) Analysis of LVEF, LVFS, LVEDd and LVEDs in CON or Ko‐Alox5 mice group received NS or DOX (*n* = 8 per group), analysed by two‐way ANOVA with Tukey's post hoc test. (L) Representative PV loops of DOX + Ko‐Alox5 or DOX mice group (*n* = 8 per group), analysed by unpaired Student's *t*‐test. (M–O) Analysis of cardiac output, +dp/dt and ‐dp/dt in CON or Ko‐Alox5 mice group received NS or DOX (*n* = 8 per group), analysed by two‐way ANOVA with Tukey's post hoc test. (P–R) Serum cTnT, CK‐MB and LDH levels in CON or Ko‐Alox5 mice groups received NS or DOX (*n* = 6 per group), analysed by two‐way ANOVA with Tukey's post hoc test. The results are shown as mean ± SEM. $*p* < 0.05: *P*‐value compared between DOX group and CON group, *p < 0.05: *p*‐value compared between DOX group and Ko‐Alox5 + DOX group.

To assess the physiological consequences of Alox5 deletion in vivo, we used homozygous Alox5‐knockout (Alox5^−/−^) mice and CON (Alox5^+/+^) mice subjected to DOX for 8 days to generate DIC models (Figure 3A). Heart homogenates from Alox5^−/−^ mice were immunoblotted with an Alox5 antibody to verify the successful ablation of Alox5 (Figure 3B). The death rates of Alox5‐deficient mice were significantly lower than those of CON mice after DOX administration (Figure 3C). Furthermore, Alox5 deletion improved body weight, HW/TL and CSA after DOX treatment (Figure 3D–F). Subsequently, the effects of Alox5 deletion on cardiac structure and function were further assessed by echocardiography and haemodynamic analysis. Importantly, we showed that Alox5 deficiency attenuated cardiac dysfunction in DIC, as indicated by increases in LVEF, LVFS, CO, +dp/dt and ‐dp/dt and reductions in LVEDd and LVEDs (Figure 3G–O). Finally, we examined the alterations in the myocardial enzyme profile after DOX administration and found Alox5 deficiency attenuated cardiac injury such as cTnT, CK‐MB and LDH (Figure 3P–R). In summary, these results indicated that Alox5 deletion attenuated myocardial injury and cardiac dysfunction in DIC.

To assess the role of Alox5 deletion in the heart in response to DOX stimulation in vitro, we silenced Alox5 by transfection with SiRNA (Si)‐Alox5 or Si‐Con (Figure S3A), as previously described [20]. Next, the efficiency of Alox5 gene transfer into the heart was evaluated by Western blotting after 8 h (Figure S3B). Furthermore, CCK‐8 experiments and LDH release assays showed a significant increase in cell viability and a substantial decrease in extracellular LDH levels in Si‐Alox5 NRVMs subjected to DOX, suggesting that Alox5 deletion attenuated cardiomyocyte death in vitro in response to DOX stimulation (Figure S3C,D). The in vivo and in vitro experiments showed that silencing Alox5 could alleviate DOX‐induced myocardial injury and cardiac dysfunction.

### 3.4 Alox5 Deletion Ameliorated Ferroptosis In Vivo

**FIGURE 4:**
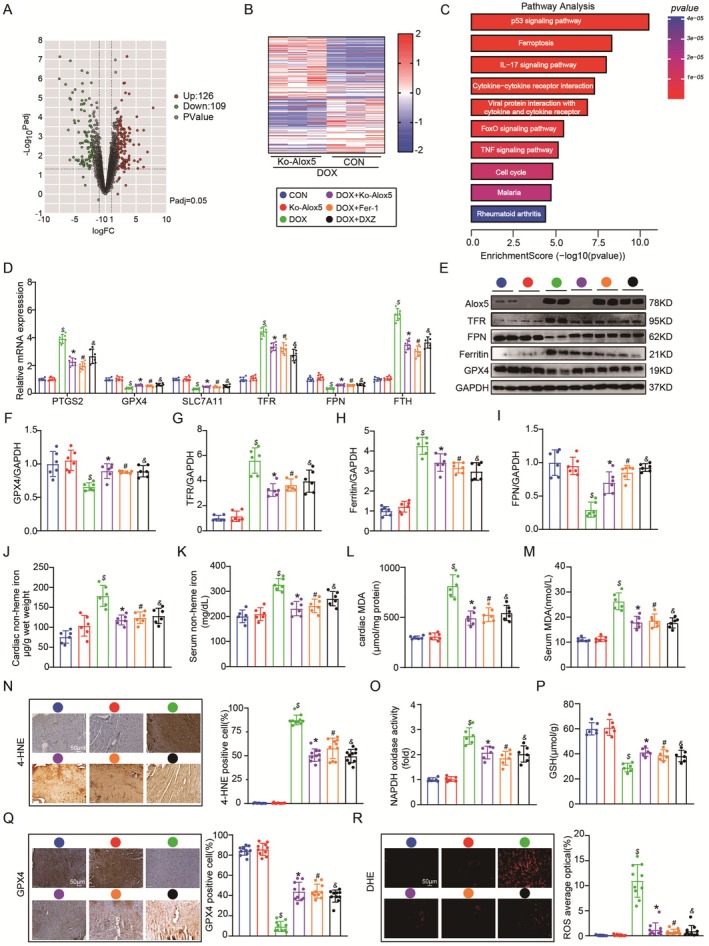
Alox5 deletion improved Ferroptosis in vivo. DOX + Ko‐Alox5 or DOX mice groups were isolated for RNA sequencing (*n* = 3 per group). (A) The volcano map was shown in DOX + Ko‐Alox5 or DOX mice group. (B) A heat map was presented in DOX + Ko‐Alox5 or DOX mice group. (C) The DEGs underwent gene ontology (GO) analyses in the DOX + Ko‐Alox5 or DOX mice group. (D) qPCR analysis of PTGS2, GPX4, SLC7A11, TFR, FPN, and FTH1 mRNA expression in CON mice group, DOX mice group, Ko‐Alox5 mice group, Ko‐Alox5 + DOX mice group, DOX + Fer‐1 mice group, or DOX + DXZ mice group (*n* = 6 per group), analysed by one‐way ANOVA with the Bonferroni post hoc test. (E–I) Representative western blots and quantitation of Alox5, TFR, FPN, Ferritin, and GPX4 expression in CON mice group, DOX mice group, Ko‐Alox5 mice group, Ko‐Alox5 + DOX mice group, DOX + Fer‐1 mice group, or DOX + DXZ mice group (*n* = 6 per group), analysed by one‐way ANOVA with the Bonferroni post hoc test. (J–M) Serum and cardiac MDA and non‐heme iron levels in CON mice group, DOX mice group, Ko‐Alox5 mice group, Ko‐Alox5 + DOX mice group, DOX + Fer‐1 mice group, or DOX + DXZ mice group (*n* = 6 per group) were analysed by one‐way ANOVA with the Bonferroni post hoc test. (N) Representative images of immunohistochemistry staining of 4‐HNE in mouse heart tissue from CON mice group, DOX mice group, Ko‐Alox5 mice group, Ko‐Alox5 + DOX mice group, DOX + Fer‐1 mice group, or DOX + DXZ mice group (*n* = 10 per group), analysed by one‐way ANOVA with the Bonferroni post hoc test. (O, P) Cardiac NAPDH and GSH level in CON mice group, DOX mice group, Ko‐Alox5 mice group, Ko‐Alox5 + DOX mice group, DOX + Fer‐1 mice group, or DOX + DXZ mice group (*n* = 6 per group), analysed by one‐way ANOVA with the Bonferroni post hoc test. (Q) Representative images of immunohistochemistry staining of GPX4 in mice heart tissue from CON mice group, DOX mice group, Ko‐Alox5 mice group, Ko‐Alox5 + DOX mice group, DOX + Fer‐1 mice group, or DOX + DXZ mice group (*n* = 10 per group), analysed by one‐way ANOVA with the Bonferroni post hoc test. (R) Representative DHE staining images and the quantitative results in CON mice group, DOX mice group, Ko‐Alox5 mice group, Ko‐Alox5 + DOX mice group, DOX + Fer‐1 mice group, or DOX + DXZ mice group (*n* = 10 per group), analysed by one‐way ANOVA with the Bonferroni post hoc test. The results are shown as mean ± SEM. $*p* < 0.05: *p*‐value compared between DOX group and CON group, *p < 0.05: *p*‐value compared between DOX group and Ko‐Alox5 + DOX group, **#** *p* < 0.05: *p*‐value compared between DOX group and DOX + Fer‐1 group, &*p* < 0.05: *p*‐value compared between DOX group and DOX + DXZ group.

According to previous studies, ferroptosis and inflammation participate in DIC [38, 41]. In addition, transcriptomics and GO enrichment analysis of adult mouse cardiomyocytes from the DOX + Ko‐Alox5 or DOX mice group showed that Alox5 deletion was similarly influenced by ferroptosis and inflammation in DIC. Compared to those in the DOX group, the ferroptosis and inflammation pathways were downregulated in the Ko‐Alox5 + DOX group (Figure 4A–C). Consistent with the transcriptomics and GO enrichment data, qPCR showed a significant decrease in ferroptosis‐associated molecules (PTGS2, TFR, and FTH1) and an increase in anti‐ferroptotic molecules (GPX4, SLC7A11, and FPN) in Alox5‐knockout mice subjected to DOX (Figure 4D), suggesting that Alox5 knockdown alleviated ferroptosis in vivo in response to DOX stimulation. Furthermore, western blotting showed a significant decrease in ferroptosis‐associated molecules (TFR and ferritin) and an increase in anti‐ferroptotic molecules (GPX4 and FPN) in Alox5‐knockout mice subjected to DOX (Figure 4E–I). Considering that abnormal iron metabolism is an important factor leading to ferroptosis, we further measured the levels of non‐heme iron in cardiac tissue and serum and found a decrease in non‐heme iron in cardiac tissue and serum in Alox5 KO mice subjected to DOX (Figure 4J,K). Lipid peroxidation could give rise to the lipid alkoxy fraction, resulting in the further production of active aldehydes, including 4‐HNE and MDA. We then examined the levels of MDA in cardiac tissue and serum and found a decrease in MDA levels in cardiac tissue and serum in Alox5 KO mice subjected to DOX (Figure 4L,M). Furthermore, immunohistochemical staining confirmed the downregulation of 4‐HNE protein expression in the cardiac tissue of Alox5 KO mice subjected to DOX (Figure 4N). GSH is involved not only in the synthesis of the cofactor GPX4 but also in the reduction of the lipid peroxide LOOH and the removal of free radicals. NADPH is the main source of synthesised GSH in vivo. Next, we further measured the levels of NADPH in cardiac tissue and found a decrease in NADPH and an increase in GSH in the cardiac tissue of Alox5 KO mice subjected to DOX (Figure 4O,P). Subsequently, immunohistochemical staining confirmed the upregulation of GPX4 protein expression levels in the cardiac tissue of Alox5 KO mice subjected to DOX (Figure 4Q). Ferroptosis ultimately occurs through the production of ROS, which damages the cell membrane and leads to cell death. Moreover, DHE staining showed that ROS were significantly increased in WT mice in response to DOX stimulation, whereas Alox5 deficiency decreased ROS levels in the heart (Figure 4R). In summary, it has been proven that Alox5 deletion ameliorates ferroptosis in vivo.

### 3.5 Alox5 Deletion Ameliorated Inflammation In Vivo

**FIGURE 5:**
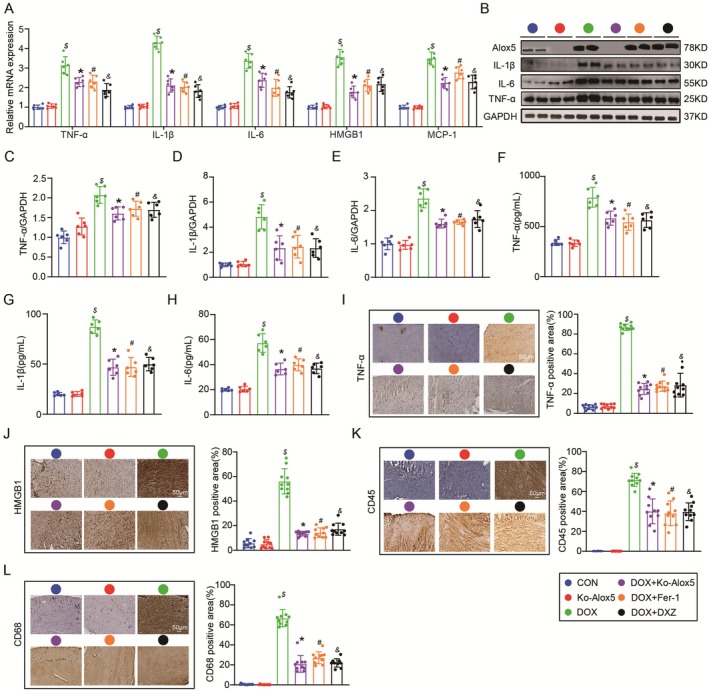
Alox5 deletion improves inflammation in vivo. (A) qPCR analysis of TNF‐α, IL‐1β, IL‐6, HMGB1 and MCP‐1 mRNA expression in CON mice group, DOX mice group, Ko‐Alox5 mice group, Ko‐Alox5 + DOX mice group, DOX + Fer‐1 mice group, or DOX + DXZ mice group (*n* = 6 per group), analysed by one‐way ANOVA with the Bonferroni post hoc test. (B–E) Representative western blots and quantitation of TNF‐α, IL‐1β and IL‐6 expression in CON mice group, DOX mice group, Ko‐Alox5 mice group, Ko‐Alox5 + DOX mice group, DOX + Fer‐1 mice group, or DOX + DXZ mice group (*n* = 6 per group), analysed by one‐way ANOVA with the Bonferroni post hoc test. (F–H) Serum TNF‐α, IL‐1β and IL‐6 levels in CON mice group, DOX mice group, Ko‐Alox5 mice group, Ko‐Alox5 + DOX mice group, DOX + Fer‐1 mice group, or DOX + DXZ mice group (*n* = 6 per group), analysed by one‐way ANOVA with the Bonferroni post hoc test. (I–L) Representative images of immunohistochemistry staining of TNF‐α, HMGB1, CD45, and CD68 in mice heart tissue samples from CON mice group, DOX mice group, Ko‐Alox5 mice group, Ko‐Alox5 + DOX mice group, DOX + Fer‐1 mice group, or DOX + DXZ mice group (*n* = 10), analysed by one‐way ANOVA with the Bonferroni post hoc test. Values represent mean ± SEM. $*p* < 0.05 indicated *p*‐value compared between DOX group and CON group, *p < 0.05 indicated *p*‐value compared between DOX group and DOX + Ko‐Alox5 group, #*p* < 0.05 indicated *p*‐value compared between DOX group and DOX + Fer‐1 group, &*p* < 0.05 indicated *p*‐value compared between DOX group and DOX + DXZ group.

Consistent with the transcriptomics and GO enrichment data, qPCR showed a significant decrease in inflammatory molecules (TNF‐α, IL‐1β, IL‐6, HMGB1 and MCP‐1) in Alox5‐knockout mice subjected to DOX (Figure 5A), suggesting that Alox5 knockdown alleviated inflammation in vivo in response to DOX stimulation. Next, Western blotting showed a significant decrease in inflammatory molecules (TNF‐α, IL‐1β and IL‐6) in Alox5‐knockout mice subjected to DOX (Figure 5B–E). We examined the alterations in inflammatory markers after DOX and found Alox5 deficiency attenuated markers of inflammation such as TNF‐α, IL‐1β and IL‐6 (Figure 5F–H). Finally, immunohistochemical staining showed that the protein expression levels of TNF‐α, HMGB1, CD45 and CD68 were increased in the DOX mice group, whereas Alox5 deficiency decreased the levels of these factors in the heart (Figure 5I–L). In summary, it has been proven that Alox5 deletion ameliorates inflammation in vivo.

### 3.6 Alox5 Deletion Ameliorated Ferroptosis and Inflammation In Vitro

**FIGURE 6:**
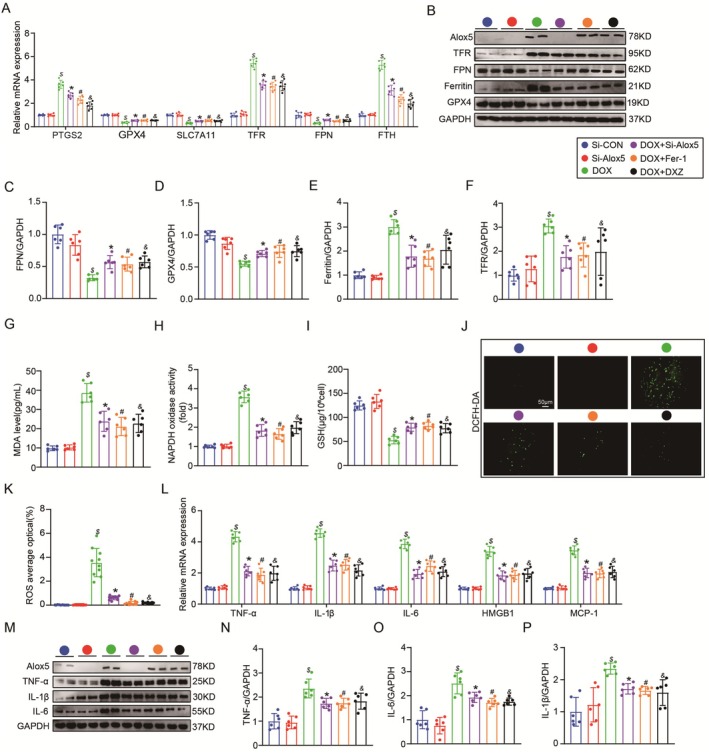
Alox5 deletion improved Ferroptosis and inflammation in vitro. (A) qPCR analysis of PTGS2, GPX4, SLC7A11, TFR, FPN and FTH1 mRNA expression in Si‐CON NRVMs group, Si‐Alox5 NRVMs group, Si‐CON+DOX NRVMs group, Si‐Alox5 + DOX NRVMs group, DOX + Fer‐1 NRVMs group, or DOX + DXZ NRVMs group (*n* = 6 per group), analysed by one‐way ANOVA with the Bonferroni post hoc test. (B–F) Representative western blots and quantitation of Alox5, TFR, FPN, Ferritin and GPX4 expression in Si‐CON NRVMs group, Si‐Alox5 NRVMs group, Si‐CON+DOX NRVMs group, Si‐Alox5 + DOX NRVMs group, DOX + Fer‐1 NRVMs group, or DOX + DXZ NRVMs group (*n* = 6 per group), analysed by one‐way ANOVA with the Bonferroni post hoc test. (G–I) The level of MDA, GSH, and NAPDH in the Si‐CON NRVMs group, Si‐Alox5 NRVMs group, Si‐CON+DOX NRVMs group, Si‐Alox5 + DOX NRVMs group, DOX + Fer‐1 NRVMs group, or DOX + DXZ NRVMs group (*n* = 6 per group) was analysed by one‐way ANOVA with the Bonferroni post hoc test. (J, K) ROS level in Si‐CON NRVMs group, Si‐Alox5 NRVMs group, Si‐CON + DOX NRVMs group, Si‐Alox5 + DOX NRVMs group, DOX + Fer‐1 NRVMs group, or DOX + DXZ NRVMs group (*n* = 10 per group), analysed by one‐way ANOVA with the Bonferroni post hoc test. (L) qPCR analysis of TNF‐α, IL‐1β, IL‐6, HMGB1 and MCP‐1 mRNA expression in Si‐CON NRVMs group, Si‐Alox5 NRVMs group, Si‐CON+DOX NRVMs group, Si‐Alox5 + DOX NRVMs group, DOX + Fer‐1 NRVMs group, or DOX + DXZ NRVMs group (*n* = 6 per group), analysed by one‐way ANOVA with the Bonferroni post hoc test. (M–P) Representative western blots and quantitation of TNF‐α, IL‐1β and IL‐6 expression in Si‐CON NRVMs group, Si‐Alox5 NRVMs group, Si‐CON + DOX NRVMs group, Si‐Alox5 + DOX NRVMs group, DOX + Fer‐1 NRVMs group, or DOX + DXZ NRVMs group (*n* = 6 per group), analysed by one‐way ANOVA with the Bonferroni post hoc test. Values represent mean ± SEM. $*p* < 0.05 indicated *p*‐value compared between Si‐CON +DOX group and Si‐CON group, *p < 0.05 indicated *p*‐value compared between Si‐CON + DOX group and DOX + Si‐Alox5 group, #*p* < 0.05 indicated *p*‐value compared between Si‐CON + DOX group and DOX + Fer‐1 group, &*p* < 0.05 indicated *p*‐value compared between Si‐CON + DOX group and DOX + DXZ group.

Alox5 deletion mainly influenced ferroptosis and inflammation in DIC in vivo. Consistent with the transcriptomics and GO enrichment data, qPCR showed a significant decrease in ferroptosis‐associated molecules (PTGS2, TFR and FTH1) and an increase in anti‐ferroptotic molecules (GPX4, SLC7A11 and FPN) in Si‐Alox5 NRVMs subjected to DOX (Figure 6A). Finally, Western blotting showed a significant decrease in ferroptosis‐associated molecules (TFR and ferritin) and an increase in anti‐ferroptotic molecules (GPX4 and FPN) in Si‐Alox5 NRVMs subjected to DOX (Figure 6B–F). Next, we measured the levels of MDA, NADPH, and GSH in NRVMs lysates and found a decrease in MDA and NADPH levels and an increase in GSH levels in Si‐Alox5 NRVMs subjected to DOX (Figure 6G–I). Consistent with the in vivo results, DCFH‐DA experiments showed that ROS were significantly increased in NRVMs in response to DOX stimulation, whereas Alox5 silencing decreased the level of ROS in NRVMs (Figure 6J,K). In summary, the in vivo and in vitro experiments showed that silencing Alox5 could ameliorate ferroptosis.

Consistent with the transcriptomics and GO enrichment data, qPCR showed a significant decrease in inflammatory molecules (TNF‐α, IL‐1β, IL‐6, HMGB1 and MCP‐1) in Si‐Alox5 NRVMs subjected to DOX (Figure 6L). In addition, Western blotting showed a significant decrease in inflammatory molecules (TNF‐α, IL‐1β and IL‐6) in Si‐Alox5 NRVMs subjected to DOX (Figure 6M–P). In summary, comprehensive in vitro and in vivo experiments showed that Alox5 deletion ameliorated inflammation.

### 3.7 DXZ Treatment Ameliorated the Deterioration of DIC Caused by Alox5 Overexpression by Inhibiting Ferroptosis

**FIGURE 7:**
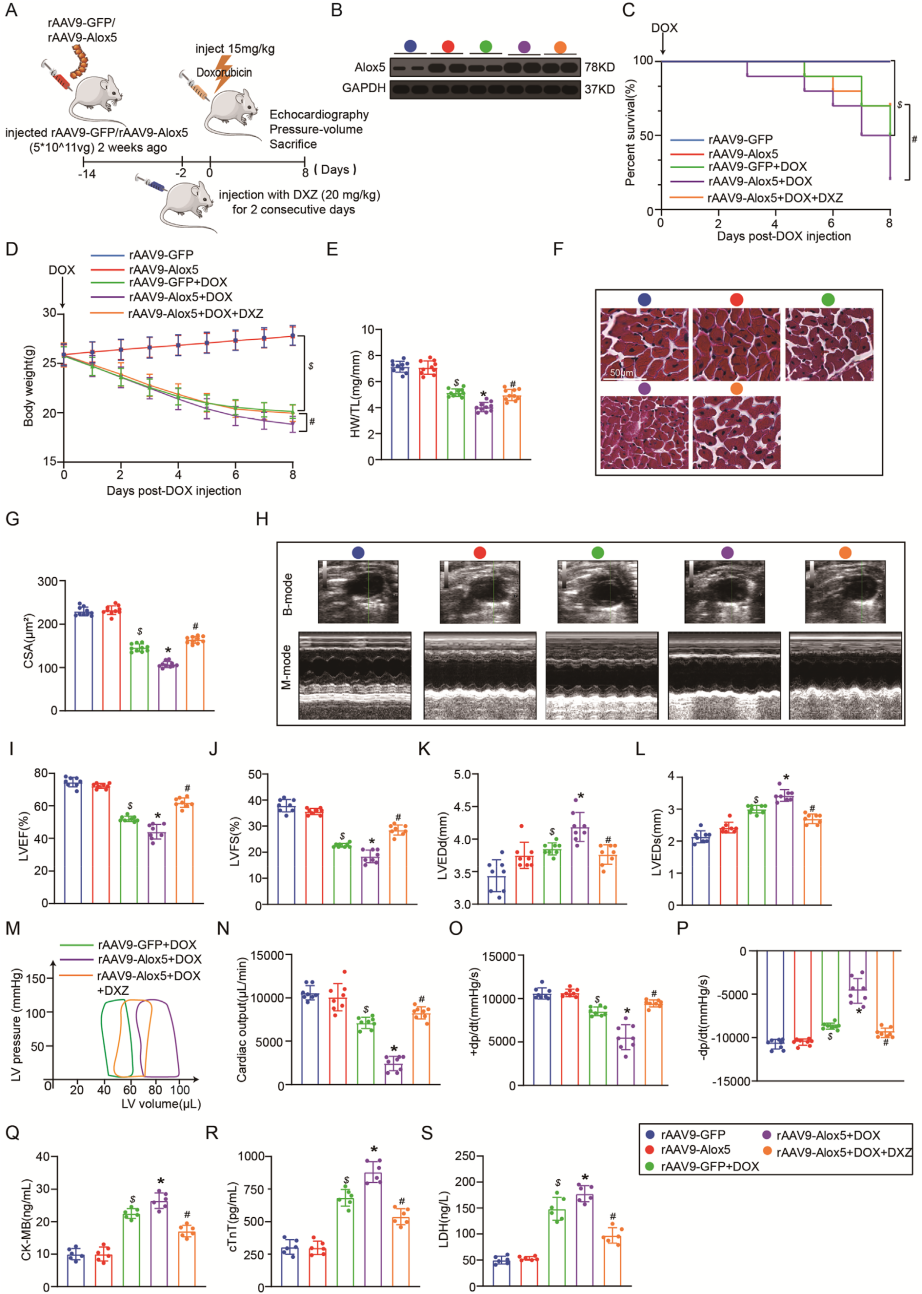
DXZ treatment ameliorated the Deterioration of DIC caused by the overexpression of Alox5 via inhibiting ferroptosis. (A) Schematic diagram of the acute mice modelling process of DIC. (B) Representative western blots of Alox5 expression in rAAV9‐GFP mice group, rAAV9‐Alox5 mice group, rAAV9‐GFP + DOX mice group, rAAV9‐Alox5 + DOX mice group, or rAAV9‐Alox5 + DOX + DXZ mice group (*n* = 6 per group). (C) Survival analysis DOX 8 day in rAAV9‐GFP mice group, rAAV9‐Alox5 mice group, rAAV9‐GFP + DOX mice group, rAAV9‐Alox5 + DOX mice group, or rAAV9‐Alox5 + DOX + DXZ mice group (*n* = 10 per group). Comparison of survival curves between DOX + rAAV9‐Alox5 and rAAV9‐Alox5 + DOX + DXZ mice group using the log‐rank (Mantel–Cox) test. (D) Body weight alterations in rAAV9‐GFP mice group, rAAV9‐Alox5 mice group, rAAV9‐GFP + DOX mice group, rAAV9‐Alox5 + DOX mice group, or rAAV9‐Alox5 + DOX + DXZ mice group (*n* = 10 per group). (E) Statistical results of the heart weight/tibia length (HW/TL) in rAAV9‐GFP mice group, rAAV9‐Alox5 mice group, rAAV9‐GFP + DOX mice group, rAAV9‐Alox5 + DOX mice group, or rAAV9‐Alox5 + DOX + DXZ mice group (*n* = 10 per group), analysed by one‐way ANOVA with the Bonferroni post hoc test. (F,G) Representative HE staining images and the quantitative results in rAAV9‐GFP mice group, rAAV9‐Alox5 mice group, rAAV9‐GFP + DOX mice group, rAAV9‐Alox5 + DOX mice group, or rAAV9‐Alox5 + DOX + DXZ mice group (*n* = 10 per group), analysed by one‐way ANOVA with the Bonferroni post hoc test. (H) Representative B‐ and M‐mode echocardiographic imaging of the heart in rAAV9‐GFP mice group, rAAV9‐Alox5 mice group, rAAV9‐GFP + DOX mice group, rAAV9‐Alox5 + DOX mice group, or rAAV9‐Alox5 + DOX + DXZ mice group (*n* = 8 per group). (I–L) Analysis of LVEF, LVFS, LVEDd and LVEDs in rAAV9‐GFP mice group, rAAV9‐Alox5 mice group, rAAV9‐GFP + DOX mice group, rAAV9‐Alox5 + DOX mice group, or rAAV9‐Alox5 + DOX + DXZ mice group (*n* = 8 per group), analysed by one‐way ANOVA with the Bonferroni post hoc test. (M) Representative PV loops of rAAV9‐Alox5 + DOX + DXZ, rAAV9‐Alox5 + DOX or rAAV9‐GFP + DOX mice group (*n* = 8 per group), analysed by one‐way ANOVA with the Bonferroni post hoc test. (N–P) Analysis of cardiac output, +dp/dt and ‐dp/dt in rAAV9‐GFP mice group, rAAV9‐Alox5 mice group, rAAV9‐GFP + DOX mice group, rAAV9‐Alox5 + DOX mice group, or rAAV9‐Alox5 + DOX + DXZ mice group (*n* = 8 per group), analysed by one‐way ANOVA with the Bonferroni post hoc test. (Q–S) Serum cTnT, CK‐MB and LDH levels in rAAV9‐GFP mice group, rAAV9‐Alox5 mice group, rAAV9‐GFP + DOX mice group, rAAV9‐Alox5 + DOX mice group, or rAAV9‐Alox5 + DOX + DXZ mice group (*n* = 6 per group) were analysed by one‐way ANOVA with the Bonferroni post hoc test. Values represent mean ± SEM. $*p* < 0.05: *p‐*value compared between rAAV9‐GFP group and rAAV9‐GFP + DOX group, *p < 0.05: *p‐*value compared between rAAV9‐Alox5 + DOX group and rAAV9‐GFP + DOX group, #*p* < 0.05: *p*‐value compared between rAAV9‐Alox5 + DOX group and rAAV9‐Alox5 + DOX + DXZ group.

To assess whether Alox5 could alleviate DIC via ferroptosis, we first injected rAAV9‐Alox5 into the tail vein, continuously injected DXZ for 2 days, and finally injected DOX for 8 days to generate a DIC model (Figure 7A). Alox5 overexpression in the heart was confirmed by Western blotting (Figure 7B). The death rates of DXZ‐treated rAAV9‐Alox5 mice were significantly lower than those of rAAV9‐Alox5 mice after DOX administration (Figure 7C). Furthermore, DXZ treatment ameliorated the deterioration of body weight, HW/TL, and CSA caused by Alox5 overexpression after DOX treatment (Figure 7D–G). Subsequently, the effects of DXZ on cardiac structure and function were assessed by echocardiography and haemodynamics analysis. Importantly, we showed that DXZ treatment attenuated cardiac dysfunction caused by Alox5 overexpression during DIC, as indicated by increases in LVEF, LVFS, CO, +dp/dt, and ‐dp/dt and reductions in LVEDd and LVEDs (Figure 7H–P). Finally, we examined the alterations in the myocardial enzyme profile after DOX and found DXZ treatment attenuated the deterioration of markers of cardiac injury such as cTnT, CK‐MB, and LDH caused by Alox5 overexpression during DIC (Figure 7Q–S). In summary, these results indicated that DXZ treatment ameliorated the deterioration of DIC caused by Alox5 overexpression by inhibiting ferroptosis.

To assess whether Alox5 could alleviate DIC through ferroptosis in vitro, we first overexpressed Alox5 by Ad‐Alox5 or Ad‐CON transfection (Figure S4A), injected DXZ for 24 h, and finally injected DOX, as previously described [34]. Next, the efficiency of Alox5 gene transfer into NRVMs was evaluated by Western blotting (Figure S4B). Furthermore, CCK‐8 experiments and LDH release assays showed a significant increase in cell viability and a reduction in extracellular LDH levels in DXZ‐treated Ad‐Alox5 NRVMs subjected to DOX, suggesting that DXZ could alleviate DOX‐induced NRVM injury caused by Alox5 overexpression by inhibiting ferroptosis (Figure S4C,D). The in vivo and in vitro experiments showed that DXZ treatment ameliorated the deterioration of DIC caused by Alox5 overexpression by inhibiting ferroptosis.

### 3.8 Alox5 Deletion Ameliorated Ferroptosis Mediated by P53

**FIGURE 8:**
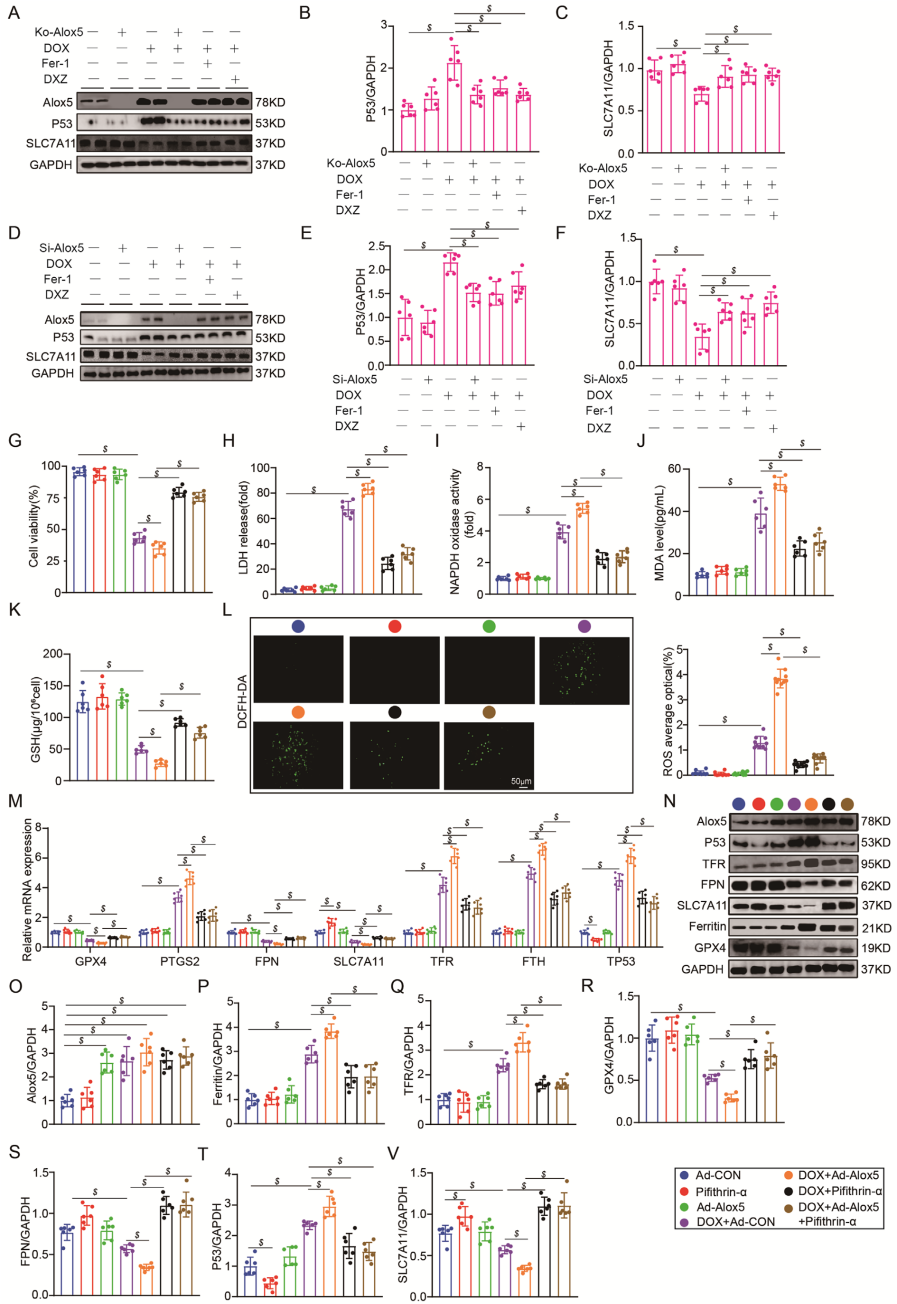
Alox5 deletion improved Ferroptosis by P53. (A–C) Representative western blots and quantitation of Alox5, P53, and SLC7A11 expression in the CON mice group, DOX mice group, Ko‐Alox5 mice group, Ko‐Alox5 + DOX mice group, DOX + Fer‐1 mice group, or DOX + DXZ mice group (*n* = 6 per group), analysed by one‐way ANOVA with the Bonferroni post hoc test. (D–F) Representative western blots and quantitation of Alox5, P53, and SLC7A11 expression in Si‐CON NRVMs group, Si‐Alox5 NRVMs group, Si‐CON + DOX NRVMs group, Si‐Alox5 + DOX NRVMs group, DOX + Fer‐1 NRVMs group, or DOX + DXZ NRVMs group (*n* = 6 per group), analysed by one‐way ANOVA with the Bonferroni post hoc test. (G) Cell viability was detected by a CCK‐8 assay in the Ad‐CON NRVMs group, Ad‐Alox5 NRVMs group, Pifithrin‐α NRVMs group, Ad‐CON+DOX NRVMs group, Ad‐Alox5 + DOX NRVMs group, DOX + Pifithrin‐α NRVMs group, or Ad‐Alox5 + DOX + Pifithrin‐α NRVMs group (*n* = 6 per group), analysed by one‐way ANOVA with the Bonferroni post hoc test. (H) LDH content in medium in Ad‐CON NRVMs group, Ad‐Alox5 NRVMs group, Pifithrin‐α NRVMs group, Ad‐CON + DOX NRVMs group, Ad‐Alox5 + DOX NRVMs group, DOX + Pifithrin‐α NRVMs group, or Ad‐Alox5 + DOX + Pifithrin‐α NRVMs group (*n* = 6 per group), analysed using one‐way ANOVA with the Bonferroni post hoc test. (I–K) The level of MDA, GSH and NAPDH in Ad‐CON NRVMs group, Ad‐Alox5 NRVMs group, Pifithrin‐α NRVMs group, Ad‐CON+DOX NRVMs group, Ad‐Alox5 + DOX NRVMs group, DOX + Pifithrin‐α NRVMs group, or Ad‐Alox5 + DOX + Pifithrin‐α NRVMs group (*n* = 6 per group) was analysed by one‐way ANOVA with the Bonferroni post hoc test. (L) ROS level in Ad‐CON NRVMs group, Ad‐Alox5 NRVMs group, Pifithrin‐α NRVMs group, Ad‐CON+DOX NRVMs group, Ad‐Alox5 + DOX NRVMs group, DOX + Pifithrin‐α NRVMs group, or Ad‐Alox5 + DOX + Pifithrin‐α NRVMs group (*n* = 10 per group), analysed by one‐way ANOVA with the Bonferroni post hoc test. (M) qPCR analysis of PTGS2, GPX4, SLC7A11, TFR, FPN, FTH1, and TP53 mRNA expression in Ad‐CON NRVMs group, Ad‐Alox5 NRVMs group, Pifithrin‐α NRVMs group, Ad‐CON+DOX NRVMs group, Ad‐Alox5 + DOX NRVMs group, DOX + Pifithrin‐α NRVMs group, or Ad‐Alox5 + DOX + Pifithrin‐α NRVMs group (*n* = 6 per group), analysed by one‐way ANOVA with the Bonferroni post hoc test. (N–V) Representative western blots and quantitation of Alox5, GPX4, P53, TFR, Ferritin, FPN and SLC7A11 expression in Ad‐CON NRVMs group, Ad‐Alox5 NRVMs group, Pifithrin‐α NRVMs group, Ad‐CON + DOX NRVMs group, Ad‐Alox5 + DOX NRVMs group, DOX + Pifithrin‐α NRVMs group, or Ad‐Alox5 + DOX + Pifithrin‐α NRVMs group (*n* = 6 per group), analysed by one‐way ANOVA with the Bonferroni post hoc test. The results are shown as mean ± SEM. $*p* < 0.05.

To further investigate how Alox5 could ameliorate DIC by inhibiting ferroptosis, transcriptomics and GO enrichment showed that Alox5 mainly affected the P53 pathway and ferroptosis pathway (Figure 4A–C). Next, Western blotting showed that Alox5 knockdown could inhibit ferroptosis to ameliorate DIC via the P53/SLC7A11 pathway in vivo and in vitro (Figure 8A–F). Subsequently, due to Alox5 overexpression‐mediated acceleration of DIC, pifithrin‐α was used to suppress the expression of P53 [36]. CCK‐8 experiments and LDH release assays showed a significant increase in cell viability and a substantial decrease in extracellular LDH levels in pifithrin‐α‐treated Ad‐Alox5 NRVMs subjected to DOX, suggesting that pifithrin‐α attenuated Ad‐Alox5‐induced cardiomyocyte death in vitro in response to DOX stimulation (Figure 8G,H). Next, we measured the levels of MDA, NADPH, and GSH in NRVMs lysates and found a decrease in MDA and NADPH and an increase in GSH in pifithrin‐α‐treated Ad‐Alox5 NRVMs subjected to DOX (Figure 8I–K). Consistent with the in vivo results, the DCFH‐DA experiment showed that ROS were significantly increased in NRVMs in response to DOX, and pifithrin‐α decreased the level of ROS caused by Alox5 overexpression in the NRVMs (Figure 8L). Consistent with the transcriptomics and GO enrichment data, qPCR showed a significant decrease in ferroptosis‐associated molecules (PTGS2, TFR, and FTH1) and an increase in anti‐ferroptotic molecules (GPX4, SLC7A11, and FPN) in pifithrin‐α‐treated Ad‐Alox5 NRVMs subjected to DOX (Figure 8M). Finally, Western blotting showed a significant decrease in ferroptosis‐associated molecules (TFR and ferritin) and an increase in anti‐ferroptotic molecules (GPX4, SLC7A11 and FPN) in pifithrin‐α‐treated NRVMs subjected to DOX or Ad‐Alox5 (Figure 8N–V). Overall, silencing Alox5 inhibited ferroptosis to ameliorate DIC via the P53/SLC7A11 pathway.

### 3.9 Pharmacological Inhibition of Alox5 Prevented DIC

**FIGURE 9:**
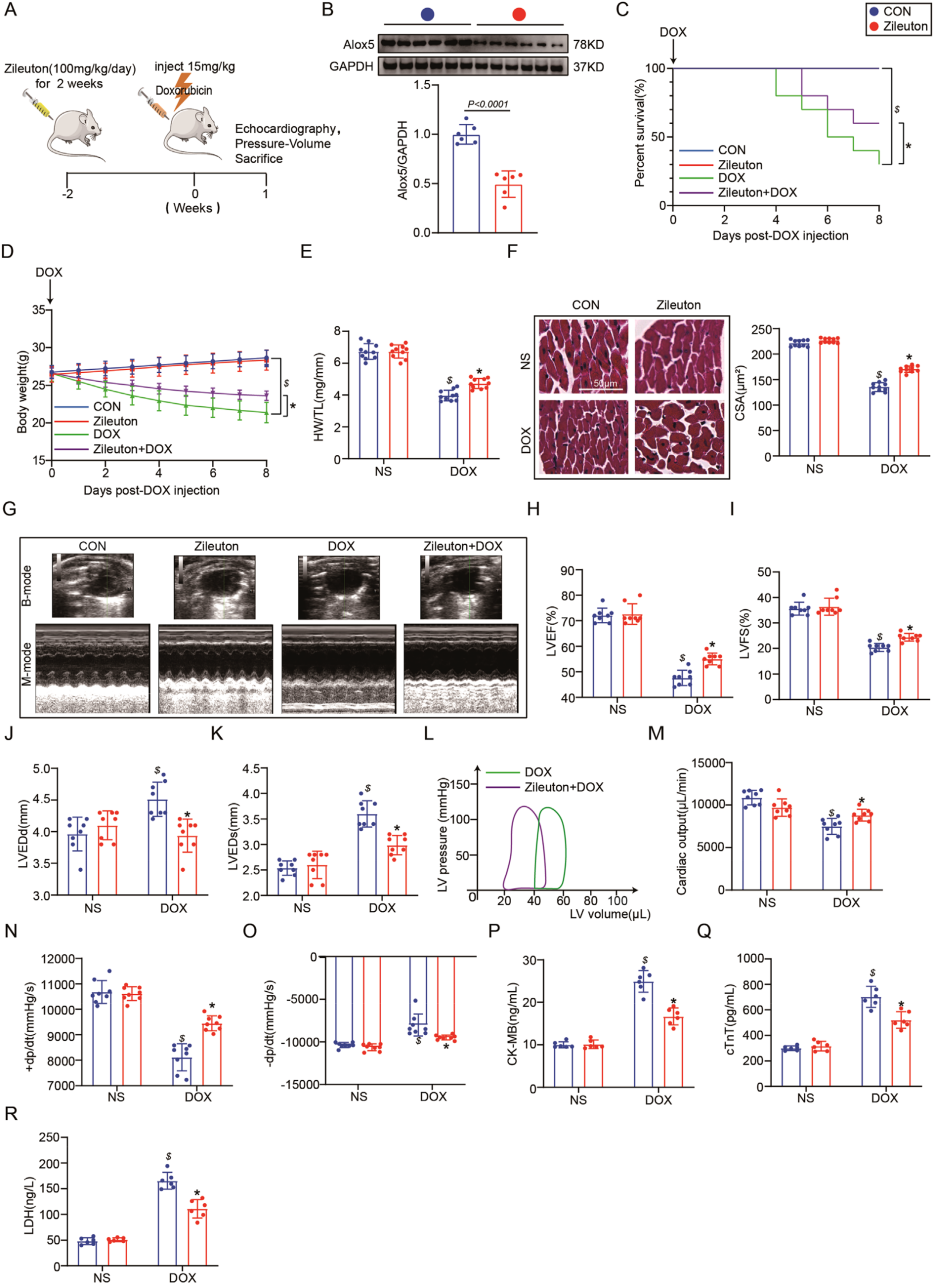
Pharmacological inhibition of Alox5 prevented DIC. (A) Schematic diagram of the acute mice modelling process of DIC. (B) Representative western blots and quantitation of Alox5 expression in CON or Zileuton‐treated mice group (*n* = 6 per group), *p*‐value between the groups marked in the histogram, analysed by unpaired Student t‐test. (C) Survival analysis DOX 8 day in CON or Zileuton‐treated mice group received NS or DOX (*n* = 10 per group). Comparison of survival curves between Zileuton+DOX and DOX mice groups using the log‐rank (Mantel–Cox) test. (D) Body weight alterations in CON or Zileuton‐treated mice group received NS or DOX (*n* = 10 per group). (E) Statistical results of the heart weight/tibia length (HW/TL) in CON or Zileuton‐treated mice group received NS or DOX (*n* = 10 per group), analysed by two‐way ANOVA with Tukey's post hoc test. (F) Representative HE staining images and the quantitative results in CON or Zileuton‐treated mice group received NS or DOX (*n* = 10 per group), analysed by two‐way ANOVA with Tukey's post hoc test. (G) Representative B‐ and M‐mode echocardiographic imaging of the heart in CON or Zileuton‐treated mice groups received NS or DOX (*n* = 8 per group). (H–K) Analysis of LVEF, LVFS, LVEDd, and LVEDs in CON or Zileuton‐treated mice group received NS or DOX (*n* = 8 per group), analysed by two‐way ANOVA with Tukey's post hoc test. (L) Representative PV loops of DOX + Zileuton or DOX mice group (*n* = 8 per group), analysed by Unpaired Student's *t*‐test. (M–O) Analysis of cardiac output, +dp/dt and ‐dp/dt in CON or Zileuton‐treated mice group received NS or DOX (*n* = 8 per group), analysed by two‐way ANOVA with Tukey's post hoc test. (P–R) Serum cTnT, CK‐MB and LDH levels in CON or Zileuton‐treated mice groups received NS or DOX (*n* = 6 per group), analysed by Two‐way ANOVA with Tukey's post hoc test. The results are shown as mean ± SEM. $*p* < 0.05: *p*‐value compared between DOX group and CON group, *p < 0.05: *p*‐value compared between Zileuton + DOX group and DOX group.

To further assess whether pharmacological inhibition of Alox5 ameliorated DIC, we used the Alox5‐specific inhibitor zileuton (Figure 9A), as previously described [35]. To determine the inhibitory effect of zileuton on cardiac Alox5, Western blotting was performed to evaluate the reduction in Alox5 protein expression in zileuton‐treated cardiac tissue (Figure 9B). The death rates of zileuton‐treated mice were significantly lower than those of CON mice after DOX administration (Figure 9C). Furthermore, zileuton improved body weight, HW/TL and CSA after DOX treatment (Figure 9D–F), which increased the possibility of its clinical use. Subsequently, the effects of zileuton on cardiac structure and function were further assessed by echocardiography and haemodynamics analysis. Importantly, we showed that zileuton attenuated cardiac dysfunction in DIC, as indicated by increases in LVEF, LVFS, CO, +dp/dt, and ‐dp/dt and reductions in LVEDd and LVEDs (Figure 9G–O). Finally, we examined the alterations in the myocardial enzyme profile after DOX administration and found zileuton improved markers of cardiac injury such as cTnT, CK‐MB, and LDH (Figure 9P–R). In summary, these results indicated that pharmacological inhibition of Alox5 could improve DOX‐induced myocardial injury and cardiac dysfunction.

To assess the effect of zileuton on the heart under DOX stimulation in vitro, we first silenced Alox5 by injecting zileuton for 24 h (Figure S5A), as previously described [35]. Next, the inhibition of Alox5 expression in NRVMs was evaluated by Western blotting (Figure S5B). Furthermore, CCK‐8 experiments and LDH release assays showed a significant increase in cell viability and a substantial decrease in extracellular LDH levels in zileuton‐treated NRVMs subjected to DOX, suggesting that zileuton attenuated cardiomyocyte death in vitro in response to DOX stimulation (Figure S5C,D). The in vivo and in vitro experiments showed that pharmacological inhibition of Alox5 could improve DOX‐induced myocardial injury and cardiac dysfunction.

## 4 Discussion

DIC is the main focus of cancer patients receiving chemotherapy [42]. Ferroptosis, which is a unique form of cell death, has been shown to be involved in DIC pathophysiology [43]. In this study, a new role of Alox5 in the regulation of DIC was found through Alox5 knockdown and overexpression experiments. Transcriptome sequencing and subsequent experiments showed that knocking down Alox5 could ameliorate DIC by inhibiting ferroptosis. Further research on the mechanism revealed that inhibiting P53 could antagonise DIC caused by Alox5 overexpression, and Alox5 could alleviate DIC by inhibiting ferroptosis through the P53/SLC7A11 pathway. In addition, pretreatment with the Alox5 inhibitor zileuton reversed DIC in mice and NRVMs. Therefore, this study identified Alox5 as a potential therapeutic target for treating DIC.

Alox5 is a non‐heme iron dioxygenase expressed in immune cells that mainly catalyses the biosynthesis of leukotrienes [18]. During ferroptosis, the oxidation of AA is mainly regulated by three enzymes: AA is activated to form AA‐CoA by ACSL4, and the activated lipid molecules undergo esterification reactions with phosphatidylcholine under the catalysis of LPCAT3 to generate AA‐PE, which is followed by lipid peroxidation under the catalysis of LOXs [10]. Structural analysis showed that an Alox5‐specific destabilising sequence was involved in orienting the carboxyl terminus, which bound the catalytic iron. In addition, stabilisation of the crystal structure of the Alox5 sequence promoted its activity through binding with FLAP [44]. Our previous studies showed that inhibiting Alox5 could improve stress overload‐induced heart failure and septic cardiomyopathy. However, our previous studies showed that both 5‐HETE and 5‐oxoETE levels were hardly changed in mice and patients with cardiac hypertrophy, suggesting that the lipoxygenase activity of Alox5 was not associated with ventricular hypertrophy following chronic pressure stress. In contrast, this study and another previous study showed that the lipoxygenase activity of Alox5 was associated with DIC and septic cardiomyopathy in mice [20, 25]. In addition, we further analysed the subcellular localisation of Alox5 in NRVMs and discovered that Alox5 expression was markedly upregulated in the nucleus during DOX stimulation. This study confirmed that DOX could increase the expression level of Alox5, but there were relevant studies indicating that DOX had a direct inhibitory action on Alox5 [45]. We previously revealed that Alox5 impaired pathological ventricular remodelling and HF independent of its enzymatic activity. However, this study revealed that Alox5 could inhibit ferroptosis to ameliorate DIC via the P53/SLC7A11 pathway. In contrast, genome‐wide screening had not shown an association between lipoxygenase and ferroptosis induced by erastin or GPX4 inhibitors, and the activation of P53 had no effect on GPX4. Secondly, researchers found that erastin did not affect the interaction between SLC7A11 and Alox12 or the enzyme activity of Alox12, and Alox12 knockout did not affect erastin‐induced ferroptosis. P53‐mediated ferroptosis was different from ferroptosis induced by erastin or GPX4 inhibitors [14]. Although Alox5 was not necessary for ferroptosis induced by common iron removers such as erastin, the loss of Alox5 expression in the context of ROS‐induced stress eliminated mutant huntingtin protein containing the expanded 94 glutamine residues (HTTQ94)‐mediated effects on ferroptosis. Interestingly, Alox5 was also necessary for HTTQ94‐mediated ferroptosis in neuronal cells in the presence of high glutamate levels. Mechanically, HTTQ94 activated Alox5‐mediated ferroptosis by stabilising FLAP, which was an important cofactor in Alox5‐mediated lipoxygenase activity. Notably, inactivation of the Alox5 gene eliminated iron removal in the striatal neurons of Huntington's disease (HD) mice. More importantly, the absence of Alox5 significantly ameliorated the pathological phenotype of these HD mice and extended their lifespan. In summary, these results demonstrated that Alox5 was crucial for mutant huntingtin (Mhtt)‐mediated ferroptosis and suggested that Alox5 was a new target for treating Huntington's disease [46]. Clausenamide directly interacted with Ser663 of Alox5, which was the PKC‐α phosphorylation site, and could prevent the nuclear translocation of Alox5, which was essential for catalysing the production of the toxic lipid 5‐HETE. Liquid chromatograph‐mass spectrometer (LC–MS)/MS‐based phospholipidomics analysis demonstrated that oxidised membrane lipids were involved in triggering ferroptotic death in dopaminergic neurons. Furthermore, the inhibition of Alox5 could significantly improve behavioural defects in a Parkinson's disease (PD) mouse model, which was confirmed to be associated with attenuating the accumulation of lipid peroxides and neuronal damage [47]. Dihydroartemisinin (DHA) had an anti‐tumour effect on pancreatic cancer cells in vitro and in vivo. DHA treatment caused ferroptosis by increasing the expression of P53 and Alox12. In addition, DHA activated anti‐tumour immunity in cancer by inhibiting myeloid‐derived suppressor cells (MDSCs) and M2 cells, as well as increasing CD8+ T cells, natural killer (NK) cells, and natural killer T (NKT) cells [48]. Firstly, ferroptosis was confirmed in neurons after spinal cord injury, and dihydroorotate dehydrogenase (DHODH) alleviated neuronal damage after spinal cord injury. Secondly, molecular evidence indicated that DHODH inhibited the activation of ferroptosis‐related molecules and reduced the production of lipid peroxides and mitochondrial damage, thereby reducing iron‐mediated neuronal death. Further analysis suggested that P53/Alox15 might be one of the mechanisms regulating DHODH. Finally, it was determined that DHODH inhibited the expression of Alox15 by inhibiting P53 [49]. Given previous research and our transcriptomics results, it was hypothesised that Alox5 could inhibit ferroptosis and alleviate DIC through the P53/SLC7A11 pathway, and this pathway must be validated in subsequent experiments.

Previous studies have shown that P53‐induced ferroptosis is a double‐edged sword [50].

### 4.1 Death‐Inducing Function of p53 in Ferroptosis

(1) Inhibition of SLC7A11 expression: the ubiquitination of ubiquitin specific peptidase 22 (USP22) could stabilise the expression of Sirtuin 1 (SIRT1). In addition, SIRT1 overexpression could lead to p53 acetylation and decreased protein levels, while p53 inhibition could increase SLC7A11 levels [51]. (2) Promotion of spermidine/spermine N1‐acetyltransferase 1 (SAT1) expression: SAT1‐induced ferroptosis required Alox15, which was a lipoxygenase that catalyses AA peroxidation. SAT1 increased the expression of Alox15, and Alox15 inhibitors rescued SAT1‐induced ferroptosis [52]. (3) Promotion of Glutamine synthase 2 (GLS2) expression: the expression of GLS2 was induced in a p53‐dependent manner in response to DNA damage or oxidative stress, and p53 was associated with the GLS2 promoter. An increase in GLS2 promoted glutamine metabolism and reduced intracellular ROS levels [53].

### 4.2 Pro‐Survival Function of p53 in Ferroptosis

(1) Inhibition of DPP4 activity. TP53 limited erastin‐induced ferroptosis by blocking dipeptidyl peptidase‐4 (DPP4) activity in a transcription‐independent manner. The absence of TP53 prevented the nuclear accumulation of DPP4, thereby promoting membrane‐related DPP4‐dependent lipid peroxidation and ultimately leading to ferroptosis [54]. (2) Promotion of CDKN1A/p21 expression: The sensitivity of ferroptosis might be regulated by stress response‐associated transcription factors and the tumour suppressor protein p53. Using CRISPR/Cas9 genome editing, small molecule probes, and high‐resolution delayed imaging, researchers found that the stability of WT p53 could delay the onset of ferroptosis caused by cystine deficiency. This delay required the p53 transcriptional target CDKN1A (encoding p21) and was associated with slow intracellular consumption of GSH and reduced accumulation of toxic lipid ROS. Therefore, the p53‐p21 axis could help cancer cells cope with metabolic stress induced by cystine deficiency by delaying the occurrence of non‐apoptotic cell death [55].

Previous transcriptomics and GO enrichment analyses first confirmed Alox5 could inhibit ferroptosis to ameliorate DIC via the P53/SLC7A11 pathway in vivo and in vitro. In the next, inhibition of P53 could ameliorate DIC caused by Alox5 overexpression by inhibiting ferroptosis. Another study showed that Alox5 was a new target gene of p53. P53 could bind to a common binding site within intron G and induce Alox5 expression in response to genotoxic factors such as Act. D or Eto. In addition, the P53 and Alox5 proteins were colocalized in the nucleus, and the Alox5 protein inhibited the transcriptional activity of p53. The coimmunoprecipitation experiment supported the direct interaction between Alox5 and p53. Alox5 might be a part of an autoregulatory negative feedback loop to limit the induction of ferroptosis genes by p53 [56].

This study had several limitations. Due to the high expression of Alox5 in macrophages, we overlooked the impact of these cells on DIC. We should conduct bone marrow transplantation and cardiomyocyte‐specific knockout mice to confirm that Alox5 was expressed in cardiomyocytes, rather than in bone marrow macrophages, and could alleviate DIC. We also overlooked gender differences and only conducted experiments on male mice. Alox5 might be related to gender differences. In addition, Alox5 not only indirectly produced LTB4 but also used other long‐chain fatty acids to create inflammation‐clearing molecules. Alox5 was indirectly involved in LTB4 biosynthesis. Alox5‐generated 5‐HETE was one of the products of Alox5 activity; thus, we overlooked its measurement. Furthermore, it was unknown how DOX upregulated the expression of Alox5. Alox5, which inhibited inflammation, and transcriptomics and GO enrichment analyses showed that Alox5 could alleviate DIC by affecting ferroptosis and inflammatory processes. It was unknown whether Alox5 mainly affected inflammatory processes to alleviate ferroptosis. Next, this experiment only improved the acute doxorubicin cardiomyopathy model, without the chronic doxorubicin cardiomyopathy model. At present, it was known that there are several small molecule inhibitors targeting Alox5, and whether these small molecule inhibitors alleviated DIC by affecting the P53/SLC7A11 signaling pathway is worth further verification. The lack of serum and myocardial samples from clinical DIC patients in this study, as well as the use of human primary cardiomyocytes, might have more clinical significance. In the meantime, this experiment did not verify whether 5‐Lox had an impact on the normal anti‐tumour effect of doxorubicin. Finally, our study did not further examine how Alox5 alleviated DIC by affecting the P53 signaling pathway.

In summary, we reported and confirmed for the first time that Alox5 inhibited ferroptosis and alleviated DIC via the P53/SLC7A11 pathway. More importantly, we found that zileuton could effectively reverse DIC. Zileuton, which was an Alox5‐specific inhibitor, was used to treat DIC, including heart failure and septic cardiomyopathy [25, 35]. This study indicated that zileuton might prevent DIC in clinical practice, which has clinical significance. However, it was still necessary to design and conduct large‐scale, large‐sample, and high‐quality clinical controlled studies to prove this finding.

## Author Contributions

**Wenxi Fang:** conceptualization (equal), data curation (equal), formal analysis (equal), investigation (equal), methodology (equal), project administration (equal), resources (equal), software (equal), validation (equal), visualization (equal), writing – original draft (equal), writing – review and editing (equal). **Zhefu Hu:** investigation (equal). **Saiyang Xie:** data curation (equal), supervision (equal), funding acquisition (equal). **Bo Shen:** funding acquisition (equal). **Xiaofeng Zeng:** investigation (equal). **Si Chen:** data curation (equal). **Shasha Wang:** investigation (equal). **Wei Deng:** funding acquisition (equal), supervision (equal).

## Ethics Statement

All animal experiments adhered to the guidelines for the Care and Use of Laboratory Animals (NIH publication number: 85‐23, revised 2011). Our experimental protocol was approved by the Guidelines for Animal Care and Use Committee of Renmin Hospital of Wuhan University (approval number: WDRM.20210902B), which complied with the Guidelines for the Care and Use of Laboratory Animals published by the US National Institutes of Health. The animal experiments were conducted at the Cardiovascular Research Institute of Wuhan University.

## Consent

All authors have read the paper and agree that it can be published.

## Conflicts of Interest

The authors declare no conflicts of interest.

## Supporting information


Data S1.

## Data Availability

The datasets generated during and/or analysed during the current study were available from the corresponding author on reasonable request.
