# Supplementary material for: Downregulation of Alox5 Inhibits Ferroptosis to Improve Doxorubicin‐Induced Cardiotoxicity via the P53/SLC7A11 Pathway
Source: J Cell Mol Med. 2025 Jun 8;29(11):e70641. doi: 10.1111/jcmm.70641 (PMC12146211; doi:10.1111/jcmm.70641)
Supplement: Supplementary file 1 — Data S1. [file JCMM-29-e70641-s001.docx]

**Supplemental Material**

**Alox5 inhibits ferroptosis to improve doxorubicin-induced cardiotoxicity via the P53/SLC7A11 pathway**

**Running title:** Alox5 inhibits doxorubicin-induced cardiotoxicity

Fang et al.

**Address correspondence to:**

Deng Wei MD, PhD

Department of Cardiology,

Renmin Hospital of Wuhan University

Jiefang Road 238, Wuhan 430060, P.R. of China.

Tel.: +86 2788041911; Fax: +86 2788066234.

Email: [vivideng1982@whu.edu.cn](mailto:vivideng1982@whu.edu.cn)

Saiyang Xie MD, PhD

Department of Cardiology,

Renmin Hospital of Wuhan University

Jiefang Road 238, Wuhan 430060, P.R. of China.

Email: [yangsai1995@whu.edu.cn](mailto:yangsai1995@whu.edu.cn)

*Requests by researchers to access the data, analytic methods, and study materials for the purposes of reproducing the results or replicating procedures can be made to the*

*corresponding author who manages the information.*

**This PDF file includes:**

- **Supplementary Tables:**

**Supplementary Table 1.** Primary antibodies used in this study.

**Supplementary Table 2.** Primers used in qPCR.

- **Supplementary Figures and Legends:**

**Supplementary Figure 1.** Alox5 expression is up-regulated in DIC in vitro.

**Supplementary Figure2.** Alox5 overexpression accelerated myocardial injury and cardiac dysfunction in DIC in vitro.

**Supplementary Figure3.** Alox5 deletion attenuated myocardial injury and cardiac dysfunction in DIC in vitro.

**Supplementary Figure4.** DXZ treatment ameliorated the deterioration of DIC caused by the overexpression of Alox5 via inhibiting ferroptosis in vitro.

**Supplementary Figure5.** Pharmacological inhibition of Alox5 prevented DIC in vitro.

**Supplementary Tables**

**Supplementary Table 1.** Primary antibodies used in this study

**Table S1. Primary antibodies used in this study**

| **Antibody** | **Customer** | **Product number** | **Dilution** | **Application** |
| --- | --- | --- | --- | --- |
| Alox5 | Abcam | Ab169755 | 1/1000 | WB |
|  |  |  | 1/100 | IF/IHC |
| TNFα | CST | 11948S | 1/1000 | WB |
|  |  |  | 1/100 | IHC |
| IL-1β | Abcam | Ab9722 | 1/1000 | WB |
| IL-6 | Abcam | Ab6672 | 1/1000 | WB |
| 4-HNE | Abcam | Ab46545 | 1/100 | IHC |
| CD45 | Abcam | Ab25603 | 1/100 | IHC |
| CD68 | Abcam | Ab125212 | 1/100 | IHC |
| P53 | CST | 2524 | 1/1000 | WB |
| GPX4 | Abcam | Ab125066 | 1/1000 | WB |
|  |  |  | 1/100 | IHC |
| β-Tubulin | CST | 2146 | 1/1000 | WB |
| β-actin | CST | 4970 | 1/1000 | WB |
| GAPDH | CST | 2118 | 1/1000 | WB |
| Ferritin | Abcam | Ab75973 | 1/1000 | WB |
| HMGB1 | Abcam | Ab79823 | 1/100 | IHC |
| Lamin B1 | Abcam | Ab65986 | 1/1000 | WB |
| SLC7A11 | CST | 12691 | 1/1000 | WB |
| FPN | Abcam | Ab85370 | 1/1000 | WB |

**Supplementary Table 2.** Primers and probes used in quantitative RT-PCR

**Table S2. Primers Used in qPCR**

| **Species** | **Gene** | **Forward Primer (5’-3’)** | **Reverse Primer (5’-3’)** |
| --- | --- | --- | --- |
| Mouse | IL-6 | GTCAGCTGGATAGCGACA | GAAGCACAGGAGCAGGTGTAGA |
| Mouse | TNF-α | GACATGCCGCCTGGAGAAAC | AGCCCAGGATGCCCTTTAGT |
| Mouse | GAPDH | ACTCCACTCACGGCAAATTC | TCTCCATGGTGGTGAAGACA |
| Mouse | Alox5 | GCAGGAAGTGGCTACTGTGGA | TGCACCCCAGTACTCATCAAG |
| Mouse | IL-1β | AGCCATGGCAGAAGTACCTG | TGAAGCCCTTGCTGTAGTGG |
| Mouse | TFR | CTCAGTTTCCGCCATCTCAGT | GCAGCTCTTGAGATTGTTTGCA |
| Mouse | FPN | GTGGAGTACTTCTTGCTCTGG | CTGCTTCAGTTCTGACTCCTC |
| Mouse | FTH | CCATCAACCGCCAGATCAAC | GAAACATCATCTCGGTCAAA |
| Mouse | GPX4 | GCCAAAGTCCTAGGAAACGC | CCGGGTTGAAAGGTTCAGGA |
| Mouse | PTGS2 | CTGCGCCTTTTCAAGGATGG | GGGGATACACCTCTCCACCA |
| Mouse | SLC7A11 | CTTTGTTGCCCTCTCCTGCTTC | CAGAGGAGTGTGCTTGTGGACA |
| Mouse | HMGB1 | ATGGGCAAAGGAGATCCTA | ATTCATCATCATCATCTTCT |
| Mouse | MCP-1 | GGAAAAATGGATCCACACCTTGC | TCTCTTCCTCCACCACCATGCAG |
| Rabbit | Alox5 | CCTACACTGTCACCGTAGCC | GCCAGTATTTGCGCTTCTCG |
| Rabbit | IL-6 | CACTTCACAAGTCGGAGGCT | AGCACACTAGGTTTGCCGAG |
| Rabbit | TNF-α | GCGGGCGGCGGTAAAATG | AGGTCCACGTCTTTGCATGT |
| Rabbit | IL-1β | GGGCGGTTCAAGGCATAACA | AAATAAATGTCACTAAAC |
| Rabbit | GAPDH | GACTCTACCCACGGCAAGTT | ATGGTGATGGGTTTCCCGTT |
| Rabbit | TFR | ACCATTGTCATATACCCGGTCA | CAATAGCCCAAGTAGCCAATCAT |
| Rabbit | FTH | TGGCAGAGACATTCCCATTTGTA | ATCAGTCATGGCCGTCTCCAG |
| Rabbit | SLC7A11 | TGGCGGTGACCTTCTCTGA | ACAAAGATCGGGACTGCTAATGA |
| Rabbit | GPX4 | CCGGCTACAATGTCAGGTTT | ACGCAGCCGTTGTTATCAAT |
| Rabbit | FPN | TAATGGGAACTGTGGCCTTC | GGAGATTATGGGGACGGATT |
| Rabbit | P53 | GTCGGCTCCGACTATACCACTATC | CTCTCTTTGCACTCCCTGGGGG |
| Rabbit | HMGB1 | AGCAATCTGAACGTCTGTCC | GTTCTTGTGATAGCCTTCGC |
| Rabbit | MCP-1 | TCTGTGCTGACCCCAATAAGGAA | GAGGTGGTTGTGGAAAAGAGAGTG |
| Rabbit | PTGS2 | GCGACTGTTCCAAACCAGCA | TGGGTCGAACTTGAGTTTGAAGTG |

**Supplementary Figures**

**Suppl Figure 1.**


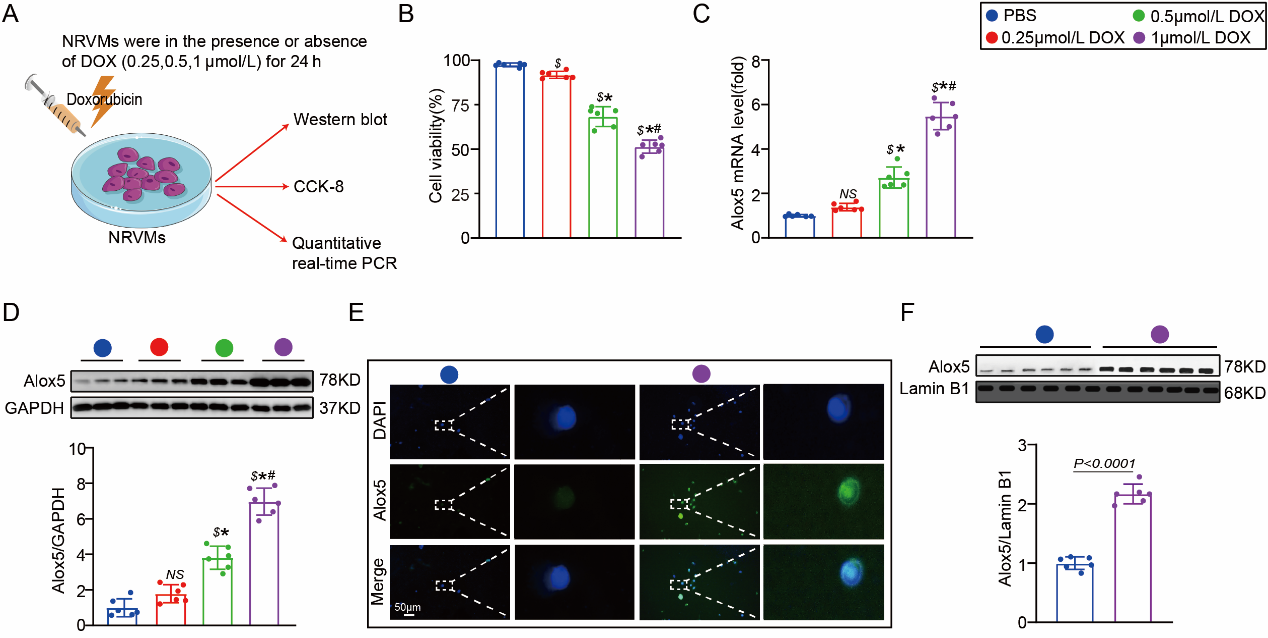


**Fig S1: Alox5 expression is up-regulated in DIC in vitro.**

**(A).** Schematic diagram of the modeling process of DOX-induced cardiomyocytes injury.

**(B).** Cell viability was detected by a CCK-8 assay in NRVMs group received PBS or DOX (n=6 per group), analyzed by one-way ANOVA with the Bonferroni post hoc test.

**(C).** qPCR analysis of Alox5 mRNA expression in NRVMs group received PBS or DOX (n=6 per group), analyzed by one-way ANOVA with the Bonferroni post hoc test.

**(D).** Representative western blots and quantitation of Alox5 expression in NRVMs group received PBS or DOX (n=6 per group), analyzed by one-way ANOVA with the Bonferroni post hoc test.

**(E).** Representative images of immunofluorescence staining of Alox5 (Green) in NRVMs group treated with PBS or 1μmol/L DOX (n=10 per group).

**(F).** Representative western blots and quantification of the Alox5 protein level in nucleus normalized to Lamin B1 protein in NRVMs group treated with PBS or 1μmol/L DOX (n=6 per group), analyzed by Unpaired Student t-test.

Values represent mean ± SEM. NS P>0.05: P value compared between 0.25μmol/L DOX group and PBS group, $P < 0.05: indicated Significantly different from PBS group, *P < 0.05: indicated Significantly different from 0.25μmol/L DOX group, #P < 0.05: indicated Significantly different from 0.5μmol/L DOX group.

**Suppl Figure 2.**

**
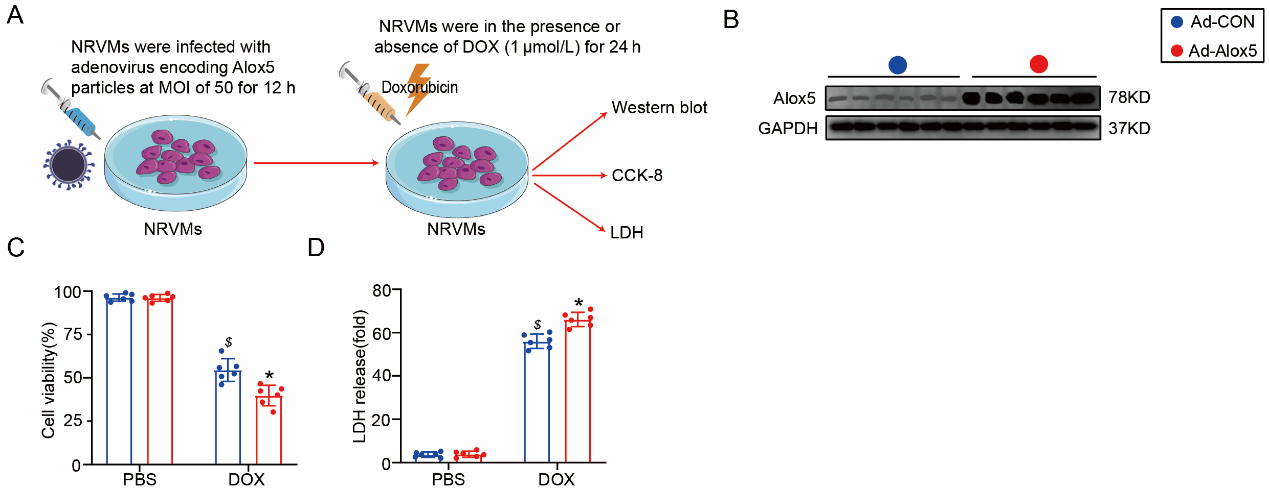
**

**Fig S2:** **Alox5 overexpression accelerated myocardial injury and cardiac dysfunction in DIC in vitro.**

**(A).** Schematic diagram of the modeling process of DOX-induced cardiomyocytes injury.

**(B).** Representative western blots of Alox5 expression in Ad-CON or Ad-Alox5 NRVMs group (n=6 per group).

**(C).** Cell viability was detected by a CCK-8 assay in Ad-CON or Ad-Alox5 NRVMs group received PBS or DOX (n=6 per group), analyzed by Two-way ANOVA with Tukey's post hoc test.

**(D).** LDH content in medium in Ad-CON or Ad-Alox5 NRVMs group received PBS or DOX (n=6 per group), analyzed by Two-way ANOVA with Tukey's post hoc test.

The results are shown as mean ± SEM. $P < 0.05: P value compared between Ad-CON+DOX group and Ad-CON group, *P < 0.05: P value compared between Ad-CON+DOX group and Ad-Alox5+DOX group.

**Suppl Figure 3.**


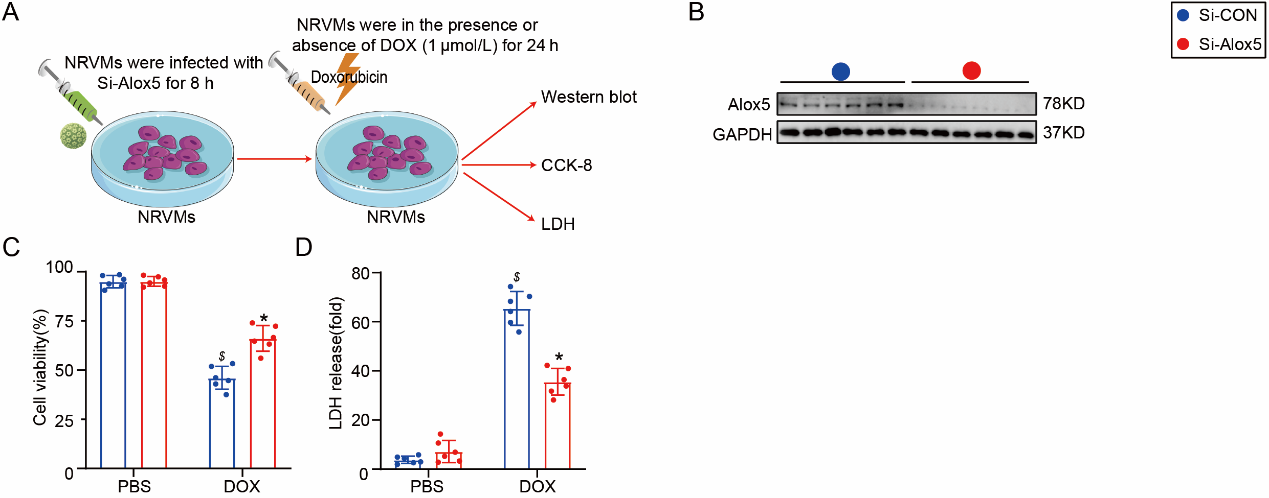


**Fig S3: Alox5 deletion attenuated myocazrdial injury and cardiac dysfunction in DIC in vitro.**

**(A).** Schematic diagram of the modeling process of DOX-induced cardiomyocytes injury.

**(B).** Representative western blots of Alox5 expression in Si-CON or Si-Alox5 NRVMs group (n=6 per group).

**(C).** Cell viability was detected by a CCK-8 assay in Si-CON or Si-Alox5 NRVMs group received PBS or DOX (n=6 per group), analyzed by Two-way ANOVA with Tukey's post hoc test.

**(D).** LDH content in medium in Si-CON or Si-Alox5 NRVMs group received PBS or DOX (n=6 per group), analyzed by Two-way ANOVA with Tukey's post hoc test.

The results are shown as mean ± SEM. $P < 0.05: P value compared between Si-CON +DOX group and Si-CON group, *P < 0.05: P value compared between Si-CON+DOX group and Si-Alox5+DOX group.

**Suppl Figure 4.**


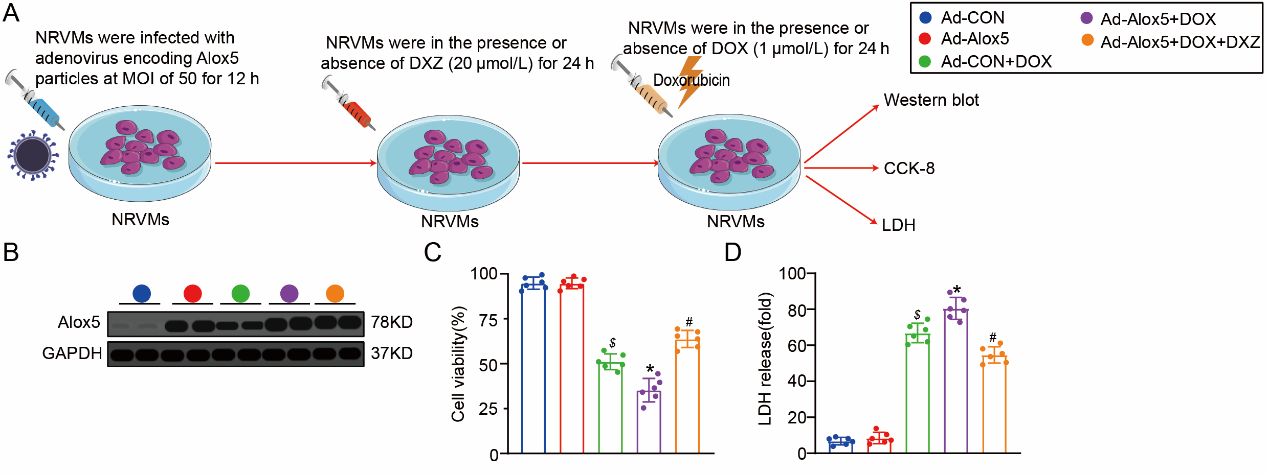


**Figure S4:** **DXZ treatment ameliorated the Deterioration of DIC caused by the overexpression of Alox5 via inhibiting ferroptosis in vitro.**

**(A).** Schematic diagram of the modeling process of DOX-induced cardiomyocytes injury.

**(B).** Representative western blots of Alox5 expression in Ad-CON NRVMs group, Ad-Alox5 NRVMs group, Ad-CON+DOX NRVMs group, Ad-Alox5+DOX NRVMs group or Ad-Alox5+DOX+DXZ NRVMs group (n=6 per group).

**(C).** Cell viability was detected by a CCK-8 assay in Ad-CON NRVMs group, Ad-Alox5 NRVMs group, Ad-CON+DOX NRVMs group, Ad-Alox5+DOX NRVMs group or Ad-Alox5+DOX+DXZ NRVMs group (n=6 per group), analyzed by one-way ANOVA with the Bonferroni post hoc test.

**(D).** LDH content in medium in Ad-CON NRVMs group, Ad-Alox5 NRVMs group, Ad-CON+DOX NRVMs group, Ad-Alox5+DOX NRVMs group or Ad-Alox5+DOX+DXZ NRVMs group (n=6 per group), analyzed by one-way ANOVA with the Bonferroni post hoc test.

Values represent mean ± SEM. $P < 0.05: P value compared between Ad-CON+DOX group and Ad-CON group, *P < 0.05: P value compared between Ad-Alox5+DOX group and Ad-CON+DOX group, **#**P < 0.05: P value compared between Ad-Alox5+DOX group and Ad-Alox5+DOX+DXZ group.

**Suppl Figure 5.**


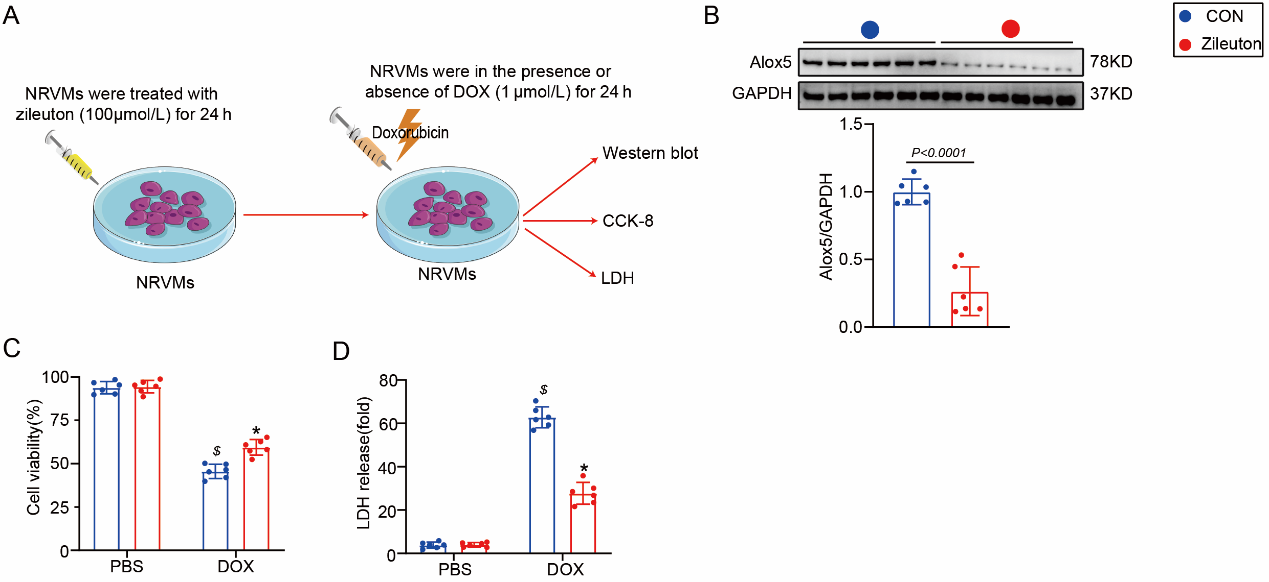


**Figure S5: Pharmacological inhibition of Alox5 prevented DIC in vitro.**

**(A).** Schematic diagram of the modeling process of DOX-induced cardiomyocytes injury.

**(B).** Representative western blots and quantitation of Alox5 expression in CON or Zileuton-treated NRVMs group (n=6 per group), P value between the groups marked in the histogram, analyzed by Unpaired Student t-test.

**(C).** Cell viability was detected by a CCK-8 assay in CON or Zileuton-treated NRVMs group received PBS or DOX (n=6 per group), analyzed by Two-way ANOVA with Tukey's post hoc test.

**(D).** LDH content in medium in CON or Zileuton-treated NRVMs group received PBS or DOX (n=6 per group), analyzed by Two-way ANOVA with Tukey's post hoc test.

The results are shown as mean ± SEM. $P < 0.05: P value compared between DOX group and CON group, *P < 0.05: P value compared between Ziluton+DOX group and DOX group.
